# Social connections and risk of incident mild cognitive impairment, dementia, and mortality in 13 longitudinal cohort studies of ageing

**DOI:** 10.1002/alz.13072

**Published:** 2023-04-27

**Authors:** Gowsaly Mahalingam, Suraj Samtani, Ben Chun Pan Lam, Darren M Lipnicki, Maria Fernanda Lima-Costa, Sergio Luis Blay, Erico Castro-Costa, Xiao Shifu, Maëlenn Guerchet, Pierre-Marie Preux, Antoine Gbessemehlan, Ingmar Skoog, Jenna Najar, Therese Rydberg Sterner, Nikolaos Scarmeas, Mary Yannakoulia, Steffi Riedel-Heller, Themis Dardiotis, Susanne Röhr, Ki-Woong Kim, Alexander Pabst, Suzana Shahar, Katya Numbers, Mary Ganguli, Tiffany F. Hughes, Chung-Chou H. Chang, Michael Crowe, Tze Pin Ng, Xinyi Gwee, Denise Qian Ling Chua, Joanna Rymaszewska, Karin Wolf-Ostermann, Anna-Karin Welmer, Jean Stafford, René Mélis, Myrra Vernooij-Dassen, Yun-Hee Jeon, Perminder S Sachdev, Henry Brodaty

**Affiliations:** 1 Centre for Healthy Brain Ageing (CHeBA), Discipline of Psychiatry and Mental Health, Faculty of Medicine and Health, UNSW Sydney, Australia; 2 School of Psychology and Public Health, La Trobe University Melbourne, UNSW Sydney, Australia; 3 Center for Studies in Public Health and Aging’ René Rachou Research Center, Oswaldo Cruz Foundation, Belo Horizonte, Minas Gerais, Brazil; 4 Department of Psychiatry, Federal University of Sao Paulo, Sao Paulo, São Paulo, Brazil; 5 Department of Geriatric Psychiatry, Shanghai Mental Health Center, Shanghai Jiaotong University School of Medicine, Shanghai, China; 6 Inserm U1094, IRD UMR270, Univ. Limoges, CHU Limoges, EpiMaCT – Epidemiology of chronic diseases in tropical zone, Institute of Epidemiology and Tropical Neurology, OmegaHealth, Limoges, France; 7 Department of Psychiatry and Neurochemistry, Neuropsychiatric Epidemiology Unit, Institute of Neuroscience and Physiology, the Sahlgrenska Academy, Centre for Ageing and Health (AGECAP), at the University of Gothenburg, Mölndal, Sweden; 8 Region Västra Götaland, Psychiatry, Cognition and Old Age Psychiatry Clinic, Sahlgrenska University Hospital, Gothenburg, Sweden; 9 1st Department of Neurology, Aiginition Hospital, National and Kapodistrian University of Athens, Athens, Greece; 10 Taub Institute for Research in Alzheimer’s Disease and the Aging Brain, The Gertrude H. Sergievsky Center, Department of Neurology, Columbia University, New York, New York, USA; 11 Department of Nutrition and Dietetics, Harokopio University, Athens, Greece; 12 Department of Neurology, University of Thessaly, Larisa, Greece; 13 Department of Neuropsychiatry, Seoul National University Bundang Hospital, Seongnam, South Korea; 14 Department of Psychiatry, Seoul National University College of Medicine, Seoul, South Korea; 15 Department of Brain and Cognitive Science, Seoul National University College of Natural Sciences, Seoul, South Korea; 16 Institute of Social Medicine, Occupational Health and Public Health (ISAP), Faculty of Medicine, University of Leipzig, Leipzig, Germany; 17 Global Brain Health Institute, Trinity College Dublin, Dublin, Ireland; 18 Health and Ageing Research Team, School of Psychology, Massey University, Palmerston, New Zealand; 19 Centre for Healthy Aging and Wellness, Faculty of Health Sciences, Universiti Kebangsaan Malaysia, Kuala Lumpur, Malaysia; 20 Departments of Psychiatry, Epidemiology, and Neurology, University of Pittsburgh, Pittsburgh, Pennsylvania, USA; 21 Youngstown State University, Youngstown, Ohio, USA; 22 Department of Medicine, School of Medicine, University of Pittsburgh, Pittsburgh, Pennsylvania, USA; 23 Department of Psychology, University of Alabama at Birmingham, Birmingham, Alabama, USA; 24 Yong Loo Lin School of Medicine, Department of Psychological Medicine, National University of Singapore, Singapore, Singapore; 25 Department of Psychiatry, Wroclaw Medical University, Wroclaw, Poland; 26 Department of Health Services and Nursing Science Research, Institute for Public Health and Nursing Research (IPP), University of Bremen, Bremen, Germany; 27 Aging Research Center & Division of Physiotherapy, Department of Neurobiology, Care Sciences and Society, Karolinska Institutet, Stockholm, Sweden; 28 MRC Unit for Lifelong Health and Ageing, University College London, London, UK; 29 Department of Geriatrics, Radboud University Medical Centre, Nijmegen, Gelderland, The Netherlands; 30 Faculty of Medical Sciences, Radboud University, Nijmegen, The Netherlands; 31 Susan Wakil School of Nursing and Midwifery, Faculty of Medicine and Health, University of Sydney, Sydney, New South Wales, Australia

**Keywords:** dementia, longitudinal, meta-analysis, mild cognitive impairment, mortality, social connections

## Abstract

**Introduction:**

Previous meta-analyses have linked social connections and mild cognitive impairment, dementia, and mortality. However, these used aggregate data from North America and Europe and examined a limited number of social connection markers.

**Methods:**

We used individual participant data (*N* = 39271, *M*_*age*_ = 70.67 (40–102), 58.86% female, *M*_*education*_ = 8.43 years, *M*_*follow-up*_ = 3.22 years) from 13 longitudinal ageing studies. A two-stage meta-analysis of Cox regression models examined the association between social connection markers with our primary outcomes.

**Results:**

We found associations between good social connections structure and quality and lower risk of incident mild cognitive impairment (MCI); between social structure and function and lower risk of incident dementia and mortality. Only in Asian cohorts, being married/in a relationship was associated with reduced risk of dementia, and having a confidante was associated with reduced risk of dementia and mortality.

**Discussion:**

Different aspects of social connections – structure, function, and quality – are associated with benefits for healthy aging internationally.

## 1 | INTRODUCTION

The 2020 Lancet Commission estimated that eliminating social isolation, one of 12 key modifiable risk factors for dementia, would reduce global dementia prevalence by 4%.^1^ Social isolation is only one component of the umbrella term social health, which encompasses an individual’s social connections, as well as their capacity and capability to interact meaningfully with others.^2,3^ Social connections are grouped into three distinct domains: structure (e.g., relationship status, living with others, frequency of interactions with friends, frequency of community group engagement), function (e.g., social support, having a confidante), and quality (e.g., relationship satisfaction, loneliness).^4,5^

Social connections are theorized to provide neuroprotection and compensation in the face of pathology.^6^ A person’s social network can influence their behavior, and have flow-on health effects.^7^ For example, social contagion theory states that health behaviors such as smoking or exercise tend to cluster within social networks.^8^ Social control, through processes such as positive reinforcement or disapproval, may also influence health behavior.^9^ Another theory states that social connections impact health via bridging and bonding pathways.^10^ The bridging pathway involves having loose ties with the community providing cognitive stimulation and promoting cognitive reserve, while bonding involves social support from close ties buffering against the harmful effects of stress.^10^ The bonding pathway involves social connection structure, function, and quality markers such as relationship status, social support, and loneliness. Loneliness is a perceived lack of social connection quality, as compared to the objective lack defining social isolation.^11^

Good social connections have been associated with lower risk of incident mild cognitive impairment (MCI),^12,13^ dementia,^14–16^ and mortality,^17–21^ and the results of numerous longitudinal studies have been subjected to meta-analysis.^13–16,21^ A meta-analysis of six low- and middle-income country (LMIC) cohorts found that loneliness was a strong predictor of MCI.^13^ One meta-analysis of 12 longitudinal studies found that living alone was associated with an elevated risk of incident dementia (risk ratio = 1.30).^14^ Another meta-analysis of 19 longitudinal studies reported that low social participation, less frequent social contact, and more loneliness, but not relationship satisfaction, were associated with incident dementia.^15^ In contrast, another meta-analysis of 31 cohort studies and 2 case-control studies found that social isolation (i.e., small social network size) and social disengagement, but not loneliness, were associated with increased risk of dementia.^16^ In a meta-analytic review of 148 studies, social isolation, living alone, and loneliness were associated with higher odds of mortality compared to obesity and these findings were consistent across sex, follow-up time, and region of the world.^21^

Previous meta-analyses of the relationship between social connectedness, MCI and dementia have several limitations. These include primarily using data from only high-income countries (HIC), principally North America and Europe, using aggregate data with estimates obtained from models accounting for different sets of covariates, and using inconsistent definitions of social connection markers. In the current study, we used meta-analytic techniques to investigate social connections and their associations with the risk of incident MCI, incident dementia, and mortality (our primary outcomes) using individual participant level data from low-, middle-, and high-income countries across six continents, harmonized social connection markers, and controlling for the same set of covariates across studies.

We hypothesized that good social connection structure (i.e., living with others, being married, frequent community group engagement, and frequent interactions with family and friends), function (i.e., social support, having a confidante), and quality (i.e., high relationship satisfaction, low levels of loneliness) would be associated with decreased risk of incident MCI, incident dementia, and mortality.

Older adults’ social connections differ across world regions. The 2017 United Nations Report on Living Arrangements of Older Persons found the proportions of older adults living alone was the highest in Europe (27%) and North America (25%), and the lowest in Asia (7%).^22^ Additionally, the number of older adults in intergenerational households was the lowest in North America (19%) and Europe (20%) and the highest in Asia (64%).^22^ Among the Organisation for Economic Cooperation and Development (OECD) countries, a lower percentage of people report having someone to rely on in Korea (79.4%) and in Japan (88.4%) than in most OECD countries (90.4%).^23^ Additionally, people socialize for fewer hours per week in Japan (4.9 h) and Korea (2 h) compared to people in most OECD countries (6 h).^23^ While the Lancet 2020 Commission attributed 4% of dementia cases worldwide to social isolation,^1^ lower population attributable fractions for social isolation have been reported for India (2%) and China (0.7%).^24^ Unlike existing meta-analyses, we ran comparative exploratory analyses to investigate ethnoregional differences in associations between social connections and the risk of incident MCI, dementia and mortality.

## 2 | METHODS

This study is presented using STROBE guidelines (Table S1). The current study is a collaborative cohort meta-analytic study, rather than a meta-analysis based on a systematic review.

### 2.1 | Ethics

This study was approved by the UNSW Human Rights Ethics Committee (HC200268). All cohort studies contributing data to this study had prior ethics approval (Table S2).

### 2.2 | Sample

Individual participant level data (*N* = 39271, *M*_*age*_ = 70.67 (40–102), 58.86% female, *M*_*education*_ = 8.43 years, *M*_*follow-up*_ = 3.22 years) were obtained from 13 longitudinal studies of ageing comprising 12 studies from the Cohort Studies of Memory in an International Consortium (COSMIC)^25^ and the English Longitudinal Study of Ageing (see Table 1 for detailed characteristics and acronyms). COSMIC member cohorts are independent studies using different methodologies and collecting different types of data (beyond the core membership requirement of cognitive data or dementia diagnoses). The cohorts included in this study were those who responded to the data call and had appropriate social connection data. All participants had at least two waves of data, and almost all were community dwelling, with a small percentage of participants from LEILA75+ (11.7% from LEILA75+; 0.38% of whole sample) in assisted living facilities.

### 2.3 | Measures

#### Social Connections Markers.

Social connections markers addressed structure (i.e., relationship status, living situation, community group engagement, interactions with family/friends), function (i.e., having a confidante, degree of social support), and quality (i.e., loneliness and relationship satisfaction); all were harmonized in accordance with previous COSMIC research.^26^ All harmonized social variables were ordinal, categorical variables except for relationship status, living situation, and having a confidante, for example, degree of social support was coded as 0 = None, 1 = Some, and 2 = Significant. Further information related to the harmonization of these variables and their coding can be found in Tables S3–S10.

Loneliness data were only available in four studies (ELSA, LEILA75+, the H70 Study, LRGS TUA), and only LRGS TUA used a validated loneliness scale (UCLA three-item loneliness scale). Similar single items for loneliness were compared across each study (Table S10). The descriptive statistics for the harmonized social connections markers can be found in Table S11.

#### Covariates.

We adjusted for age, sex, and education at baseline in partially adjusted models featuring all 13 cohorts, and additionally for depression, history of diabetes, hypertension, smoking, and cardiovascular risk at baseline in fully adjusted models featuring the 10 cohorts with these data. All covariates were harmonized in accordance with previous COSMIC research^4^ (Tables S12–S16). The descriptive statistics for the harmonized covariates can be found in Table S17.

#### Outcome Variables.

Our primary outcome variables were the risk of all-cause incident MCI, incident dementia, and mortality. MCI was classified as scoring at least 1.5 SD below the mean of a cognitively normal sample on one or more cognitive tests, and without a current diagnosis of dementia.^27^ Not all studies captured subjective complaints, a core diagnostic feature of MCI,^28^ so we were unable to include this criterion. Descriptive statistics and standardization of cognitive tests addressing global cognition and cognitive domains (i.e., memory, language, executive functioning, perceptual motor, and attention/processing speed) used are described in Tables S18–S19 and Text S1. Dementia was identified using consensus diagnoses or, where unavailable, established cutoff scores for cognitive tests (see Table S20).

### 2.4 | Statistical analyses

All statistical analyses were conducted using *R* software^29^ and *R* packages *survival*^30^ for Cox regression models, *mice*^31^ for multiple imputation, and *metafor*^32^ for meta-analyses.

We conducted two-stage individual participant level data (IPD) meta-analyses^33^ to pool estimates across studies. In the first stage, Cox regressions^34^ were conducted for incident MCI, incident dementia, and mortality with each social connection marker, adjusting for age, sex, and education within each study.

These Cox regression models were used to determine hazard ratios (HRs) for MCI, dementia, and mortality (see Table S21). We used time in study (years) to compute event times and adjusted for age, sex, and education at baseline. The proportional hazards assumption was satisfied via Schoenfeld residuals. Participants’ mortality data were censored at the final study wave if dying after this. Fully adjusted mortality models were run with eight cohorts. Sensitivity analyses (partially and fully adjusted models) were conducted using cause-specific models for incident MCI and dementia using cohort studies with mortality data.

We explored ethnoregional differences between Western (ELSA, The H70 Study, HELIAD, LEILA75+, MAS, MYHAT, PREHCO) and Asian (CLAS, KLOSCAD, LRGS TUA, SLAS) cohorts using subgroup meta-analyses. With only single studies from Africa and South America, we did not include these in our ethnoregional analyses.

In the second stage, we used a random-effects meta-analysis with a restricted maximum likelihood estimator (REML) to obtain a weighted average of the HRs from all studies. Heterogeneity was measured using *τ*^2^ and *I*^2^, and publication bias using Egger’s test and funnel plots.

#### Missing data.

Under the missing at random assumption, missing data for covariates and social connections markers with fewer than 50% missing data were imputed using multiple imputation by chained equations, which was informed using auxiliary variables.^35,36^ The imputation process produced 20 imputed data sets and results were pooled using Rubin’s rules.^36^ Patterns of missing data were inspected visually to confirm that missing data were related to auxiliary variables and to reduce the impact of non-random missingness on the results.

## 3 | RESULTS

Baseline demographic and other characteristics of the cohort studies are summarized in Table 1. Incident MCI, incident dementia, and mortality rates after baseline are presented in Table 2.

### 3.1 | Associations between social connections and incident MCI, dementia, and mortality

**FIGURE 1:**
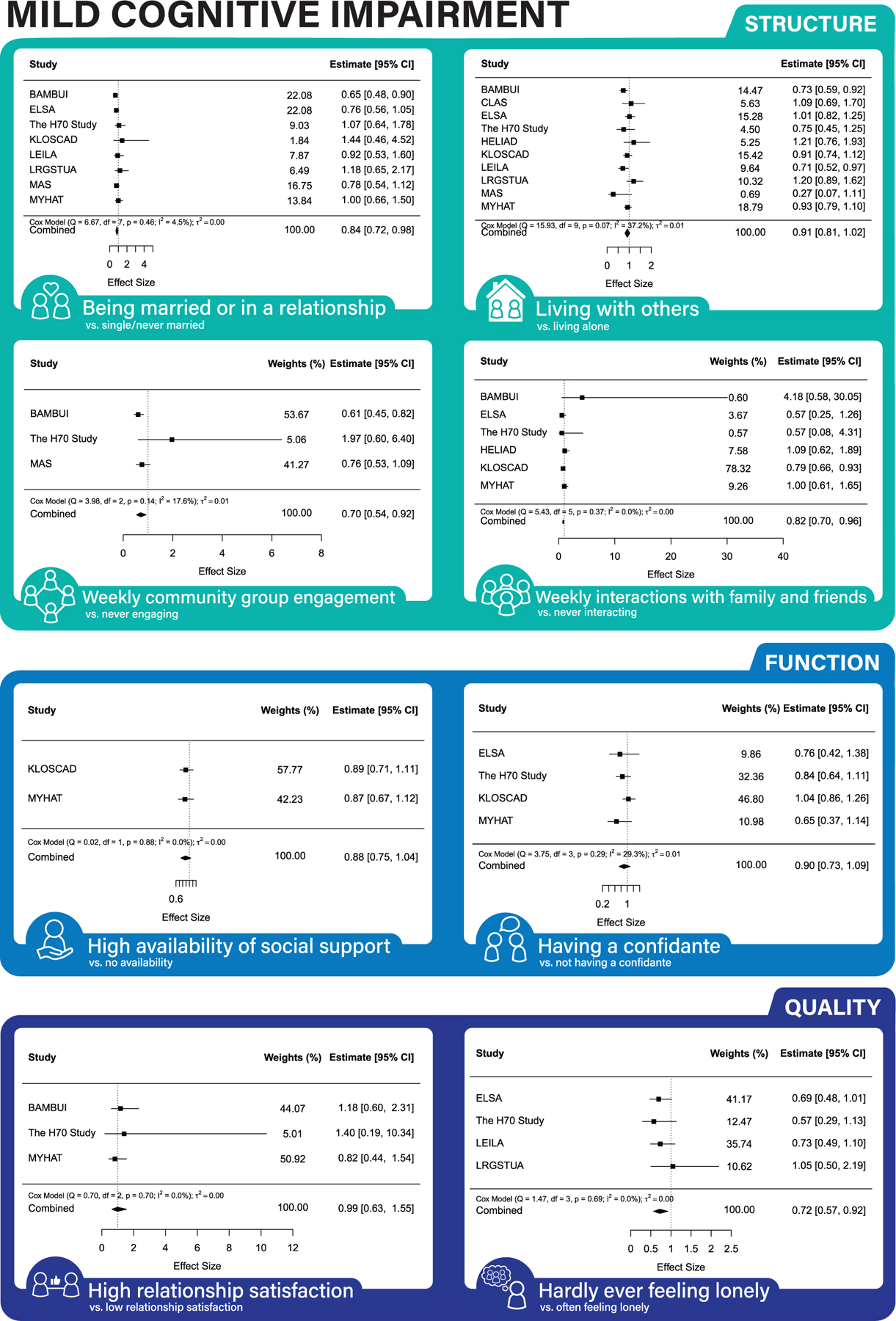
Association between social connection markers and incident MCI (fully-adjusted models).

**FIGURE 2:**
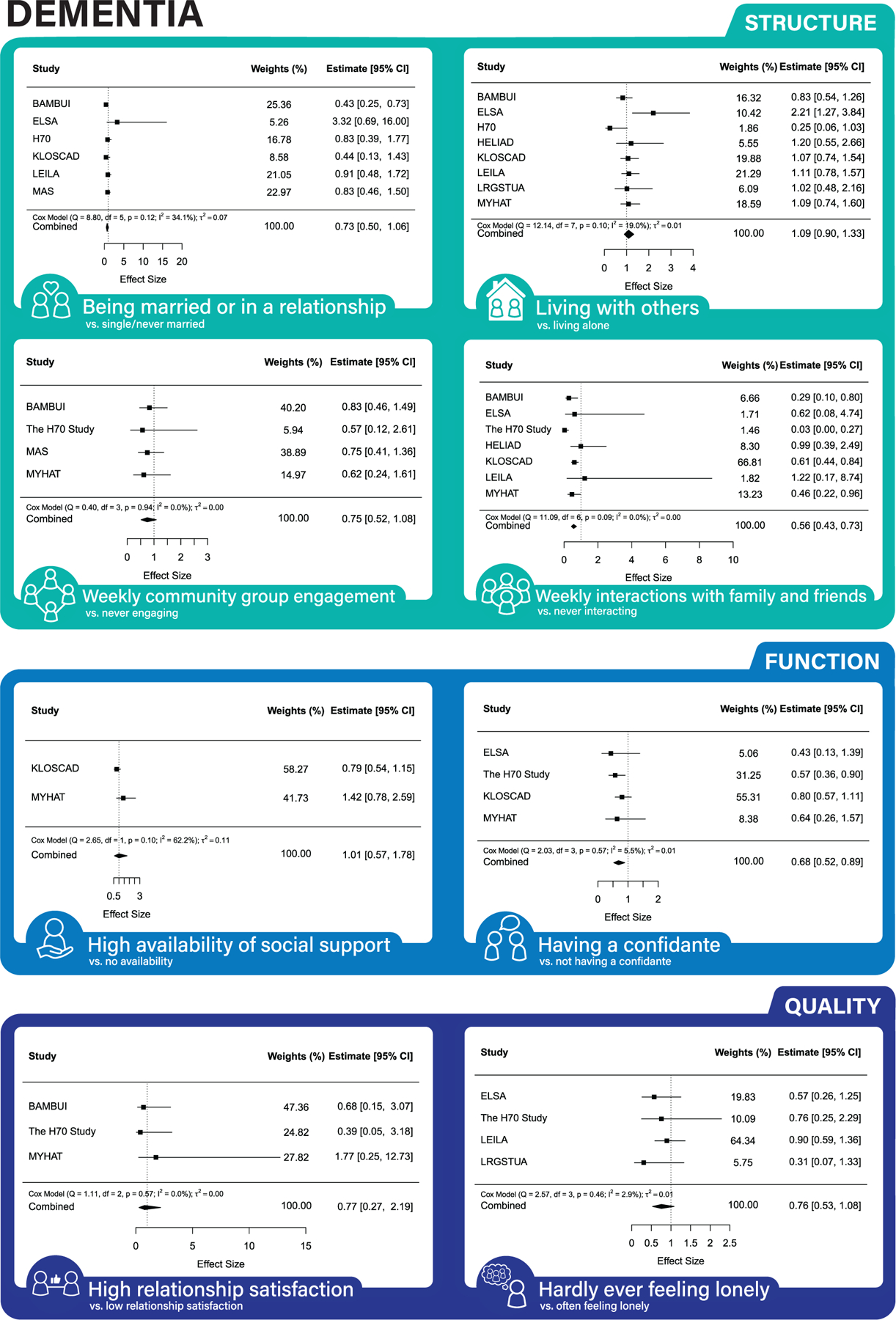
Association between social connection markers and incident dementia (fully-adjusted models).

**FIGURE 3:**
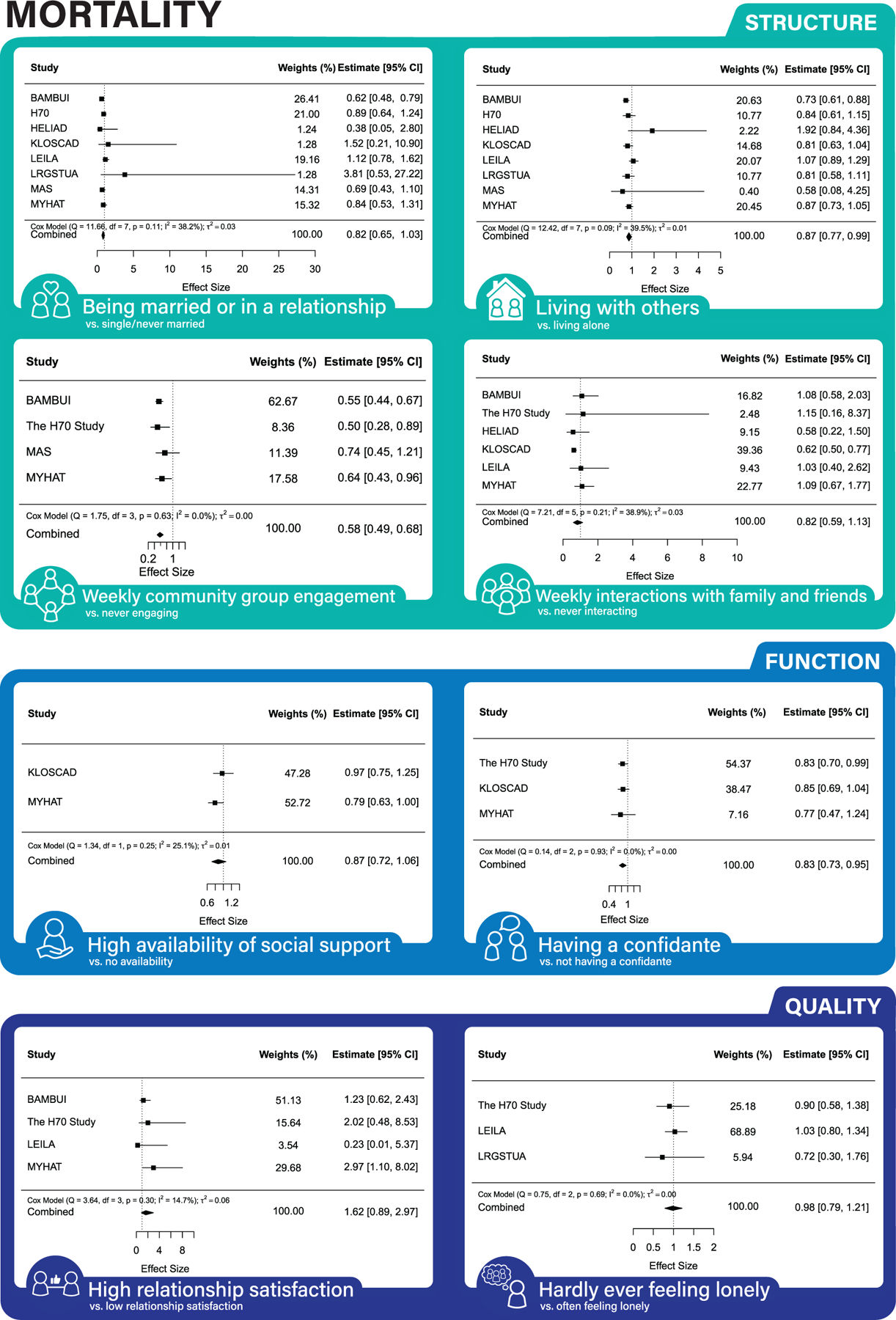
Association between social connection markers and mortality (fully-adjusted models).

Across both partially and fully adjusted models (see Figures 1, 2, and 3 for forest plots, Figures S1–S3 for funnel plots, and Table 3 for results), being married/in a relationship, weekly community group engagement, weekly interactions with friends/family, and never feeling lonely were associated with lower MCI risk. Monthly/weekly interactions with friends/family and having a confidante were associated with lower dementia risk. Living with others, yearly/monthly/weekly community engagement, and having a confidante were associated with lower mortality risk.

Sensitivity analyses (cause-specific models), as presented in Table S22, largely replicated the main model results, exceptions being married/in a relationship and incident MCI (not replicated in the partially or fully adjusted models); and never feeling lonely and incident MCI (replicated only in the partially adjusted models).

Some results were inconsistent between the partially and fully adjusted models. Results found only in the partially adjusted models were monthly interactions with family and friends and a high degree of social support being associated with a decreased risk of MCI; never feeling lonely decreasing the risk of dementia; being married/in a relationship, monthly, weekly interactions with family and friends, and high degree of social support decreasing the risk of mortality. Results found only in the fully adjusted models were being married/in a relationship, and weekly engagement with community groups were associated with lower risk of MCI and having a confidante with lower risk of mortality.

**FIGURE 4:**
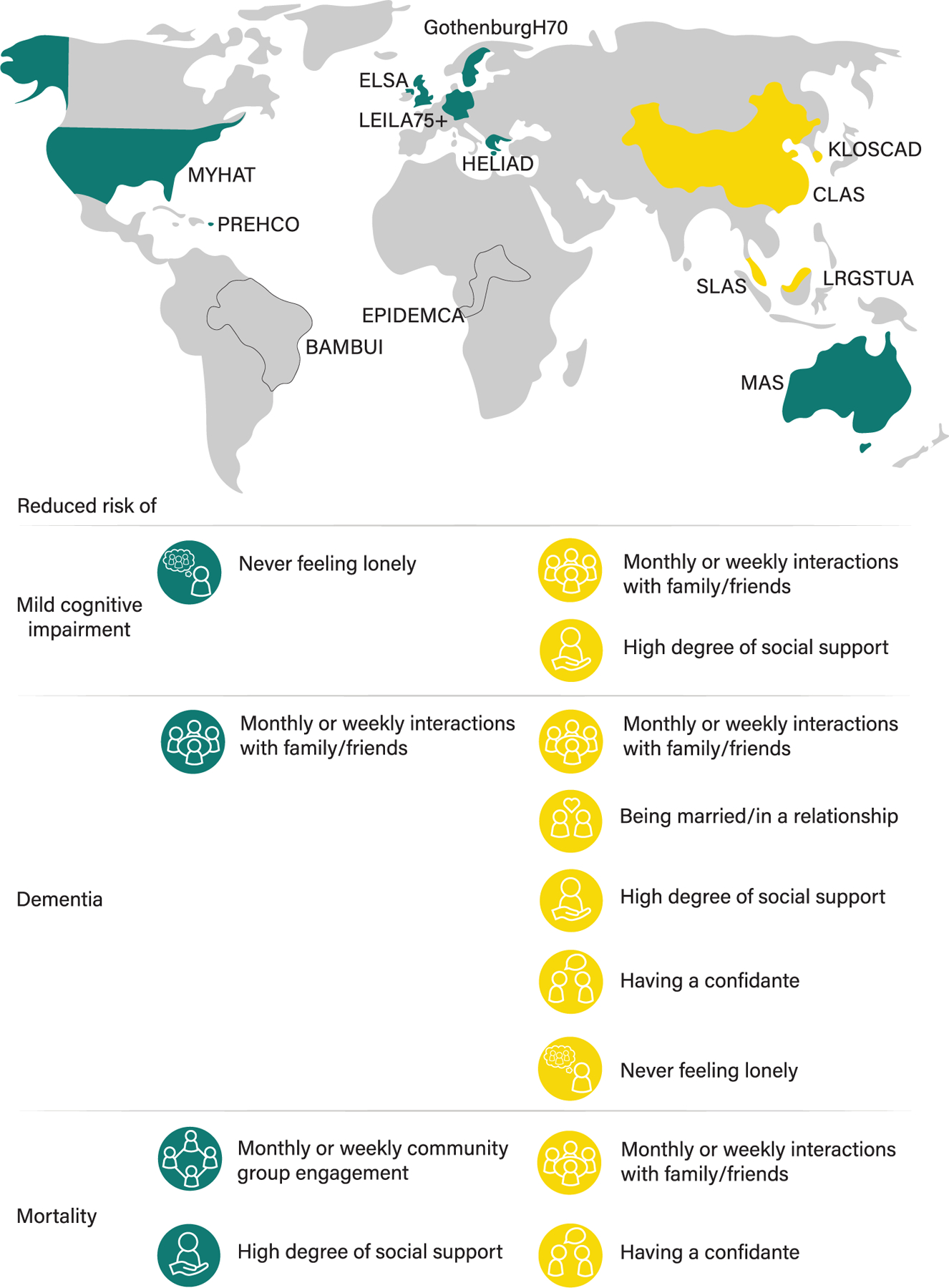
Association between social connection markers and mortality (partially-adjusted models) within Asian and Western cohorts.

Although estimates of heterogeneity were low (*I*^2^ = 0.00–34.02%), we explored ethnoregional differences between Asian and Western countries in the associations between social connection markers and each of MCI, dementia, and mortality. Figure 4 shows the significant estimates within each set of cohorts. We estimated each association separately for both subgroups and compared these estimates (see Table S23). While estimates for Asian cohorts were not significantly different from those in Western cohorts, some effects were significant *within* one set of cohorts but not the other. Estimates significant *only* in Asian cohorts were weekly interactions with family and friends and a high degree of social support reducing risk of MCI; being married or in a relationship, a high degree of social support, having a confidante and never feeling lonely reducing risk of dementia; and monthly/weekly interactions with family and friends and having a confidante reducing risk of mortality. Estimates significant only in Western cohorts were never feeling lonely reducing risk of MCI; monthly/weekly community group engagement, and high degree of social support reducing risk of mortality. For both Western and Asian cohorts, monthly/weekly interactions with family and friends reduced risk of dementia.

## 4 | DISCUSSION

We investigated the associations between social connection markers and incident MCI, incident dementia and mortality in 13 longitudinal studies of ageing. The results support our hypotheses that all three outcomes are negatively associated with social connection structure (i.e., being in a relationship/married, living with others, frequent interactions with family/friends, and frequent community group engagement), function (i.e., social support, having a confidante), and quality (i.e., high relationship satisfaction, never feeling lonely) markers.

Good social connections, that is, structure and quality, were associated with lower risk of incident MCI. Specifically, lower risk of MCI was associated with being married/in a relationship, weekly community group engagement, weekly interactions with family and friends and never feeling lonely. The association between regular community group engagement, interactions with friends/family and lower risk may partially be explained by higher levels of physical activity,^37^ social contagion of protective health behaviors,^7,8^ or the stress buffering effect of close relationships.^10^ Our findings on interactions with family and friends are consistent with a previous study that found frequent phone contact with family and friends reduced odds of MCI.^38^ Additionally, the association between loneliness and MCI confirmed the findings of two previous studies.^12,13^ We did not find an association between social connection function and MCI risk. Previous research shows lower levels of social support for people with MCI compared to cognitively healthy individuals^39^ but there is a lack of longitudinal studies examining the association between low social support and risk of incident MCI over time.

Similarly, good social connection structure (monthly/weekly interactions with family and friends) and function (having a confidante) were associated with lower risk of dementia. Similar to the pathways for reducing risk of MCI, having frequent contact with family and friends may promote protective health behaviors such as physical activity through social control.^7,9,37^ The association between having a confidante and lower dementia risk supports the hypothesized bonding pathways between social connections and cognitive health, whereby close ties are thought to play a stress buffering role via the neuroendocrine and hypothalamus-pituitary-adrenal axis (HPA) axis function.^10^ Contrary to our hypothesis, we did not find an association between social connection quality and risk of dementia, which mirrors findings in one meta-analysis,^16^ but not in another.^15^ Given that we found loneliness was associated with risk of MCI, and previously that it is associated with cognitive decline,^4^ it is possible that loneliness needs early targeting to affect the course of dementia progression. The effects of loneliness on dementia progression may be reduced through the maintenance of high social reserve, which involves the ability to form and maintain meaningful social relationships.^40^

In line with our hypothesis, social connection structure and function were associated with decreased risk of mortality. Our findings regarding structural facets of social connections including living with others and community group engagement confirms results of previous studies.^17–21^ As with dementia, however, we did not find an association between social connection quality and mortality. This contrasts with other meta-analyses that found loneliness was associated with increased risk of mortality^20,21^; however, these studies used continuous loneliness scales rather than the single-item loneliness questions to which we were restricted.

Poor social connections may increase mortality risk via multiple mechanisms. In the UK Biobank cohort study, excess mortality risk was associated with poor social connection structure, whereas loneliness was associated with socioeconomic status, unhealthy behaviors, poor mental health, and poor self-rated health.^41^ A recent systematic review highlighted multiple meta-analyses which found associations between social connections, poor biological health, poor psychological health, poor lifestyle, and increased morbidity and mortality.^5^ This includes poor social connections being associated with elevated activity of the sympathetic nervous system and altered function of the HPA (glucocorticoid resistance), which interact and promote chronic inflammation, potentially leading to multiple health conditions.^5^ For instance, in the MIDUS study, social support provided by high-quality close ties, such as marriage, were associated with a lower composite score of biological risk related to cardiovascular functioning, HPA axis activity, inflammation, nervous system functioning, and metabolism.^42^

Ethnoregional comparisons revealed that certain estimates were significant *within* a set of either Western or Asian cohorts, but these estimates were not significantly different *between* Western versus Asian cohorts. Being married/in a relationship reduced risk of dementia only in Asian cohorts, which may be related to greater stigma and ostracism related to being unmarried in Asian culture.^43^ Monthly or weekly community group engagement reduced risk of mortality only in Western cohorts, which may be related to fewer people per household in Western countries^22^ and seeking diversity in types of social connections.^44^ Frequent interactions with family and friends reduced risk of MCI, dementia, and mortality in Asian cohorts, but only reduced risk of dementia in Western cohorts. Given that community group engagement reduced risk of mortality in Western cohorts, it may be that people in Western countries receive health benefits via structured community activities rather than informal interactions. Structured community group activities such as exercise groups may provide not only social but cognitive and physical stimulation, which is related to the bridging pathway of social connections increasing cognitive reserve.^10^ Having a confidante reduced risk of dementia and mortality only in Asian cohorts. In Asian countries, there is a sense of shame related to asking for emotional support,^45^ and overcoming this stigma may have benefits for those who confide in others. High degree of social support and never feeling lonely did not have a consistent pattern of results, which may be due to a low number of cohorts with available data resulting in wide confidence intervals. These results should be interpreted with caution, as there is significant heterogeneity within Asian cohorts.

The possibilities for reverse causality should also be considered, despite our longitudinal design. It may be that cognitive and physical difficulties may have already impacted social connections at baseline in our study. Given the decades-long build-up of neuropathology in the case of MCI/dementia and initial symptoms such as social withdrawal or depression, individuals may become socially isolated and less connected over time. Physical conditions leading eventually to death may also be related to symptoms such as fatigue and social withdrawal. Other people may also begin to distance themselves from people experiencing cognitive or physical difficulties who become depressed or experience difficulties participating in social activities.

### 4.1 | Strengths

Our study used individual participant data instead of aggregate data typically used in meta-analyses. This approach provides detailed information at the participant level and allows us to control for the same set of covariates across studies, enabling comparisons of estimates between these studies. We used a large sample of 13 longitudinal cohort studies of ageing and while previous studies used data primarily from North America and Europe, we also included data from South America, Africa, Asia, and Australia. Further, we investigated a wide range of social connection markers. We found no significant differences between seven Western and four Asian cohort studies in the associations between social connections and risks of MCI, dementia or mortality. We examined the social connection markers separately rather than combining them into composite scores for social connection structure, function, and quality. While markers are correlated to some extent, examining each marker individually allows us to make specific recommendations about the type and amount of social connections required to reduce one’s risk of dementia or mortality. Additionally, many studies focusing on dementia incidence do not account for the possibility of dying before experiencing MCI/dementia, which can result in biased estimates of associations between risk factors and dementia. We ran sensitivity analyses (cause-specific models) for incident MCI and dementia using studies with mortality data and observed mostly similar results.

### 4.2 | Limitations

This study was a collaborative cohort study using data from ELSA and COSMIC studies which answered to the data call, rather than a systematic review with a meta-analysis. While a systematic review meta-analysis is the gold standard for *aggregate data* meta-analyses, it would be impractical to obtain access to, harmonize and analyze *raw individual participant data* from all relevant longitudinal ageing studies. Notably, our funnel plots did not show evidence of publication bias. The harmonization of data in our study prevented analysis using highly detailed measures of social connections, as we were limited by studies lacking such data. We could only use algorithmic classification of MCI that did not consider subjective cognitive complaints, as consistent data for these or consensus MCI diagnoses were lacking a across studies. Despite removing participants with dementia or MCI at baseline in the relevant models, reverse causality may have influenced our results given a relatively shorter mean follow-up time of 3.22 years (range 0–16 years). As the cohort studies had more social connection structure markers, than social connection function and quality markers, the results may reflect a conceptual bias. We were unable to compare with African and South American cohorts due to the lack of multiple cohorts from these continents.

### 4.3 | Future directions

Our study clarifies which social connection markers are associated with reduced risk of MCI, dementia, and mortality. Future studies may further explore the causal pathways (such as bridging and bonding, social contagion, or social control) from social connection markers to cognitive, mental, and physical health. The use of validated scales (rather than single questions) would allow more fine-grained analyses and identification of minimum thresholds of social connections for promoting cognitive reserve and physical health. Additional work is required to understand the social health ‘capacity to meaningfully engage with others’, as we currently lack a theoretical understanding of, and assessments for, this component of social health. The next steps would be to determine whether interventions to improve social health can change cognitive trajectories.

Our results lead to recommendations for maintaining social connections for healthy ageing. Social prescribing by doctors, geriatricians, and allied health may help middle-aged and older adults in the community to reduce their risk of dementia or mortality.^46^ Examples could be encouraging older adults to engage in weekly interactions with friends or family, or in weekly community engagement, and or to live with others (including in intergenerational households).

## 5 | CONCLUSIONS

Harmonized individual participant level data from 13 longitudinal cohort studies of ageing support the associations between good social connections and lower risk of incident MCI, incident dementia, and mortality.

## Supplementary Material

supinfo

## Figures and Tables

**TABLE 1:** Cohort characteristics—demographic variables.

Study	Country	Sample size	Baseline and final assessment waves	Age at baseline (SD)	Sex (% Female)	Years of education at baseline (SD)
Bambui Cohort Study of Ageing (BAMBUI)^38^	Brazil	1602	1997, 2011	69.3 (7.40)	60.05	2.70 (2.95)
Chinese Longitudinal Study of Ageing (CLAS)^39^	China	3059	2011, 2018	71.11 (7.86)	54.23	8.36 (5.34)
English Longitudinal Study of Ageing (ELSA)^40^	England	9300	2014, 2016–17	66.72 (9.54)	55.39	11.43 (1.82)
Epidemiology of Dementia in Central Africa African (EPIDEMCA)^41^	Central Republic and Republic of Congo	2001	2011–12, 2015	73.68 (6.71)	61.82	1.92 (3.81)
Gothenberg H70 Birth Cohort Studies (the H70 study)^42^	Sweden	1221	2000–04, 2009–11	75.29 (5.69)	78.38	9.58 (4.09)
Hellenic Longitudinal Investigation of Ageing & Diet (HELIAD)^43^	Greece	2032	2009–16, 2013–18	73.12 (5.75)	60.13	8.05 (5.04)
Korean Longitudinal Study on Cognitive Aging and Dementia (KLOSCAD)^44^	South Korea	6749	2010–12, 2017–18	70.46 (7.08)	57.34	8.2 (5.37)
Leipzig Longitudinal Study of the Aged (LEILA75+)^45^]	Germany	1263	1996–98, 2011–14	82.48 (5.30)	76.25	11.93 (1.79)
Neuroprotective Model for Healthy Longevity among Malaysian Older Adults Towards Using Ageing (LRGS TUA)^46^]	Malaysia	2322	2013–14, 2018–19	69.05 (6.23)	52.02	5.14 (3.99)
Sydney Memory and Ageing Study (MAS)^47–50^	Australia	1037	2005–07, 2011–14	78.84 (4.82)	55.16	11.60 (3.47)
Monongahela-Youghiogheny Healthy Aging Team (MYHAT)^48^	USA	1919	2006–08, 2017–18	77.66 (7.45)	60.97	10.85 (2.40)
Puerto Rican Elderly Health Conditions Study (PREHCO)^49^	Puerto Rico	3962	2002–03, 2006–07	71.71 (8.47)	59.84	7.84 (4.65)
Singapore Longitudinal Study of Ageing (SLAS)^50^	Singapore	2804	2003–05, 2007–09	66.02 (7.69)	63.16	6.64 (4.60)
Overall	N/A	39271	N/A	70.67 (8.73)	58.86	8.43 (4.91)

*Note*: Sample size includes participants with MCI or Dementia at baseline (except for CLAS, ELSA).

**TABLE 2:** Annual Incidence of MCI, dementia, and mortality (per 1000 people per year) for each study.

Study	Country	Annual incidence of MCI per 1000*	Annual incidence of dementia per 1000*	Annual incidence of mortality per 1000*
Bambui Cohort Study of Ageing (BAMBUI)	Brazil	68.5	11.3	49.8
Chinese Longitudinal Study of Ageing (CLAS)	China	134	26.3	N/A
English Longitudinal Study of Ageing (ELSA)	England	34.5	5.6	N/A
Epidemiology of Dementia in Central Africa (EPIDEMCA)	Central African Republic and Republic of Congo	23	27.9	66.6
Gothenberg H70 Birth Cohort Studies (the H70 study)	Sweden	78.1	19.7	88.7
Hellenic Longitudinal Investigation of Ageing & Diet (HELIAD)	Greece	104	19.6	24.5
Korean Longitudinal Study on Cognitive Aging and Dementia (KLOSCAD)	South Korea	53.4	10	24.5
Leipzig Longitudinal Study of the Aged (LEILA75+)	Germany	136	52.8	105
Neuroprotective Model for Healthy Longevity among Malaysian Older Adults Towards Using Ageing (LRGS TUA)	Malaysia	182	8.37	41.7
Sydney Memory and Ageing Study (MAS)	Australia	136	22.6	32.5
Monongahela-Youghiogheny Healthy Aging Team (MYHAT)	USA	117	11.9	48.9
Puerto Rican Elderly Health Conditions Study (PREHCO)	Puerto Rico	48.2	3.42	38.6
Singapore Longitudinal Study of Ageing (SLAS)	Singapore	36.2	11.5	18.0
Overall	N/A	70.3	12.6	46

* *Note*: These rates are not age-adjusted.

**TABLE 3:** Cox regression model results including partially and fully adjusted models.

Outcome	Social connection marker	Partially adjusted models				Fully adjusted models			
		HR (95%CI)	*I*^2^ (%)	*τ* ^2^	Egger’s test^b^	HR (95%CI)	*I*^2^ (%)	*τ* ^2^	Egger’s test
MCI	**Married/in a relationship**	0.86 (0.73, 1.02)	23.76	0.02	*z* = 2.02, p = 0.04	**0.84 (0.72, 0.98)**	4.67	0.00	z = 2.12, p = 0.03
	Living with others	0.91 (0.82, 1.01)	35.95	0.01	z = −0.76, p = 0.44	0.91 (0.81, 1.02)	38.13	0.01	z = −0.88, p = 0.38
	Community Group Engagement^a^								
	Yearly	0.90 (0.73, 1.12)	0.00	0.00	z = 1.66, p = 0.10	0.86 (0.68, 1.09)	0.00	0.00	z = 1.47, p = 0.14
	Monthly	1.00 (0.82, 1.23)	8.68	0.01	z = 0.42, p = 0.68	1.05 (0.81, 1.35)	15.75	0.01	z = 0.96, p = 0.34
	**Weekly**	0.76 (0.56, 1.03)	42.01	0.05	z = 1.35, p = 0.18	**0.70 (0.54, 0.92)**	17.48	0.01	z = 1.90, p = 0.06
	Interactions with family/friends^a^								
	Yearly	0.90 (0.62, 1.30)	0.00	0.00	z = 1.15, p = 0.25	0.94 (0.62, 1.42)	0.00	0.00	z = 1.15, p = 0.25
	**Monthly**	**0.85 (0.72, 0.99)**	0.00	0.00	z = 0.09, p = 0.93	0.87 (0.73, 1.04)	3.31	0.00	z = 0.75, p = 0.45
	**Weekly**	**0.82 (0.71, 0.95)**	0.00	0.00	z = 0.90, p = 0.37	**0.82 (0.70, 0.96)**	0.01	0.00	z = 0.99, p = 0.32
	**High degree of Social Support**	**0.83 (0.71, 0.97)**	0.00	0.00	z = −0.06, p = 0.95	0.88 (0.75, 1.04)	0.00	0.00	
	Having a confidante	0.89 (0.72, 1.11)	65.56	0.04	z = −1.76, p = 0.08	0.90 (0.73, 1.09)	29.33	0.01	z = −1.72, p = 0.09
	High relationship Satisfaction	0.95 (0.62, 1.46)	0.00	0.00	z = 0.01, p = 1.00	0.99 (0.63, 1.55)	0.00	0.00	z = 0.41, p = 0.69
	**Never feeling lonely**	**0.62 (0.50, 0.77)**	0.00	0.00	z = 0.48, p = 0.63	**0.72 (0.57, 0.92)**	0.00	0.00	z = 0.34, p = 0.73
Dementia	Married/in a relationship	0.76 (0.52, 1.11)	35.94	0.11	z = 1.99, p = 0.05	0.73 (0.50, 1.06)	34.07	0.07	z = 1.29, p = 0.20
	Living with others	1.08 (0.90, 1.30)	20.09	0.02	z = −0.03, p = 0.98	1.09 (0.90, 1.33)	19.03	0.01	z = −1.04, p = 0.30
	Community Group Engagement^a^								
	Yearly	0.98 (0.69, 1.40)	0.00	0.00	z = −0.34, p = 0.73	0.97 (0.67, 1.40)	0.00	0.00	z = −0.15, p = 0.88
	Monthly	0.81 (0.57, 1.14)	0.00	0.00	z = −0.40, p = 0.69	0.83 (0.58, 1.20)	0.00	0.00	z = −0.08, p = 0.93
	Weekly	0.76 (0.54, 1.07)	0.00	0.00	z = −0.02, p = 0.99	0.75 (0.52, 1.08)	0.00	0.00	z = −0.56, p = 0.58
	Interactions with family/friends^a^								
	Yearly	0.87 (0.54, 1.42)	0.00	0.00	z = 0.73, p = 0.47	0.82 (0.45, 1.51)	0.00	0.00	z = 0.62, p = 0.53
	**Monthly**	**0.48 (0.35, 0.66)**	0.00	0.00	z = −0.46, p = 0.64	**0.49 (0.34, 0.69)**	0.00	0.00	z = −0.87, p = 0.38
	**Weekly**	**0.53 (0.41, 0.67)**	0.00	0.00	z = −0.79, p = 0.43	**0.56 (0.43, 0.73)**	0.00	0.00	z = −1.14, p = 0.26
	High degree of Social Support	0.83 (0.47, 1.46)	54.29	0.13	z = −0.68, p = 0.50	1.01 (0.57, 1.78)	62.21	0.11	
	**Having a confidante**	0.68 (0.46, 1.02)	60.51	0.11	z = −0.21, p = 0.83	**0.68 (0.52, 0.89)**	5.53	0.01	z = −1.03, p = 0.30
	High relationship Satisfaction	0.68 (0.27, 1.70)	0.00	0.00	z = 1.19, p = 0.23	0.77 (0.27, 2.19)	0.00	0.00	z = 0.06, p = 0.95
	**Never feeling lonely**	**0.63 (0.40, 0.99)**	34.02	0.07	z = −2.06, p = 0.04	0.76 (0.53, 1.08)	2.94	0.01	z = −1.37, p = 0.17
		**HR (95%CI)**	*I*^2^ (%)	*τ* ^2^	Egger’s test^b^	**HR (95%CI)**	*I*^2^ (%)	*τ* ^2^	Egger’s test
Mortality	**Married/in a relationship**	**0.78 (0.66, 0.93)**	19.08	0.01	z = −0.45, p = 0.65	0.82 (0.65, 1.03)	38.21	0.03	z = 0.96, p = 0.34
	**Living with others**	0.91 (0.81, 1.01)	37.48	0.01	z = 0.54, p = 0.59	**0.87 (0.77, 0.99)**	39.48	0.01	z = 0.63, p = 0.53
	Community Group Engagement^a^								
	**Yearly**	**0.78 (0.66, 0.93)**	0.00	0.00	z = −0.63, p = 0.53	**0.81 (0.68, 0.96)**	0.02	0.00	z = −0.43, p = 0.67
	**Monthly**	**0.61 (0.52, 0.72)**	0.00	0.00	z = 0.06, p = 0.95	**0.67 (0.57, 0.80)**	0.00	0.00	z = 0.44, p = 0.66
	**Weekly**	**0.52 (0.44, 0.61)**	0.00	0.00	z = 0.84, p = 0.40	**0.58 (0.49, 0.68)**	0.00	0.00	z = 0.65, p = 0.52
	Interactions with family/friends^a^								
	Yearly	0.77 (0.50, 1.17)	25.03	0.05	z = 1.84, p = 0.07	0.86 (0.44, 1.70)	50.09	0.18	z = 1.49, p = 0.14
	**Monthly**	**0.80 (0.66, 0.96)**	0.00	0.00	z = 0.87, p = 0.38	0.91 (0.66, 1.26)	40.02	0.06	z = 1.13, p = 0.26
	**Weekly**	**0.70 (0.56, 0.88)**	20.21	0.02	z = 1.15, p = 0.25	0.82 (0.59, 1.13)	38.89	0.06	z = 0.90, p = 0.37
	**High degree of Social Support**	**0.79 (0.67, 0.93)**	0.00	0.00	–	0.87 (0.72, 1.06)	25.14	0.01	–
	**Having a confidante**	0.88 (0.73, 1.05)	60.05	0.02	z = −0.81, p = 0.42	**0.83 (0.73, 0.95)**	0.00	0.00	z = −0.33, p = 0.74
	High relationship Satisfaction	1.34 (0.85, 2.13)	0.00	0.00	z = −0.90, p = 0.37	1.62 (0.89, 2.97)	14.73	0.06	z = −0.78, p = 0.43
	Never feeling lonely	0.97 (0.77, 1.22)	20.69	0.01	z = −1.36, p = 0.17	0.98 (0.79, 1.21)	0.00	0.00	z = −0.86, p = 0.39

*Note*: The reference groups for the social connection markers were single/never married (vs. married/in a relationship), living alone (vs. living with others), never engaging in community activities (vs. yearly/monthly/weekly community group engagement), never interacting with family/friends (vs. yearly/monthly/weekly interactions with family/friends), low degree of social support (vs. high degree of social support), not having a confidante (vs. having a confidante), low relationship satisfaction (vs. high relationship satisfaction), and often feeling lonely (vs. never feeling lonely).

## Data Availability

All aggregate participant data are presented either in the manuscript or appendix. Individual participant data cannot be made publicly available because they are protected by a confidentiality agreement. Data were provided by the contributing studies to COSMIC on the understanding and proviso that the relevant study leaders be contacted for further use of their data and additional formal data sharing agreements be made. Researchers can apply to use COSMIC data by completing a COSMIC Research Proposal Form available from https://cheba.unsw.edu.au/consortia/cosmic/research-proposals.

## Highlights

- Social connection structure (being married/in a relationship, weekly community group engagement, weekly family/friend interactions) and quality (never lonely) were associated with lower risk of incident MCI.
- Social connection structure (monthly/weekly friend/family interactions) and function (having a confidante) were associated with lower risk of incident dementia.
- Social connection structure (living with others, yearly/monthly/weekly community group engagement) and function (having a confidante) were associated with lower risk of mortality.
- Evidence from 13 longitudinal cohort studies of ageing indicates that social connections are important targets for reducing risk of incident MCI, incident dementia, and mortality.
- Only in Asian cohorts, being married/in a relationship was associated with reduced risk of dementia, and having a confidante was associated with reduced risk of dementia and mortality.

## RESEARCH IN CONTEXT

1. **Systematic Review**: We searched for relevant meta-analyses using platforms such as Web of Science and PsycINFO. Social connections are associated with increased risk of MCI, dementia, and mortality. Most meta-analytic research relies on aggregate estimates from North America and Europe.
2. **Interpretation**: Our results reveal that good social connections (structure, function, and quality) reduced risk of incident MCI, dementia, and mortality. Being married/in a relationship reduced risk of dementia. Having a confidante reduced risk of dementia and mortality only in Asian cohorts.
3. **Future Directions**: Our study highlights the association between good social connections and preservation or enhancement of cognitive and physical health. We need further research on mechanisms such as bridging and bonding pathways, social contagion and social control to better understand how social connections impact health. We also need to develop interventions aimed at enhancing and preserving the social connections of older adults.
