# Supplementary material for: Social connections and risk of incident mild cognitive impairment, dementia, and mortality in 13 longitudinal cohort studies of ageing
Source: Alzheimers Dement. Author manuscript; Available in PMC 2024 Nov 1. (PMC10603208; doi:10.1002/alz.13072)
Supplement: supinfo [file NIHMS1892749-supplement-supinfo.docx]

Table of Contents

[Table S1. STROBE statement checklist for cohort studies 2](#_Toc128906364)

[Table S2. Ethics approvals for each cohort study contributing to the current study’s dataset. 5](#_Toc128906365)

[Table S3. Relationship Status Harmonisation 6](#_Toc128906366)

[Table S4. Living Situation Harmonisation 7](#_Toc128906367)

[Table S5. Social Interactions with Friends/Family Harmonisation 8](#_Toc128906368)

[Table S6. Community Group Engagement Harmonisation 9](#_Toc128906369)

[Table S7. Degree of Support Harmonisation 10](#_Toc128906370)

[Table S8. Someone to Confide In/Emotional Support Harmonisation 11](#_Toc128906371)

[Table S9. Relationship Satisfaction Harmonisation 12](#_Toc128906372)

[Table S10. Loneliness Frequency (reverse scored) Harmonisation 13](#_Toc128906373)

[Table S11. Characteristics of Sample- Social Connections 14](#_Toc128906374)

[Table S12. Smoking Harmonisation 17](#_Toc128906375)

[Table S13. Diabetes Mellitus Harmonisation 18](#_Toc128906376)

[Table S14. Hypertension Harmonisation 19](#_Toc128906377)

[Table S15. Cardiovascular Disease Harmonisation 20](#_Toc128906378)

[Table S16. Depression Harmonisation 21](#_Toc128906379)

[Table S17. Characteristics of Sample- Covariates 22](#_Toc128906380)

[Table S18. Cognitive tests used to determine mild cognitive impairment and their descriptive statistics at baseline. 23](#_Toc128906381)

[Table S19. Percentage of missing data at baseline wave for global cognition and all cognitive domains. 24](#_Toc128906382)

[Supplementary Text S1. Standardization of cognitive outcome scores 25](#_Toc128906383)

[Table S20. Identification of people with dementia 26](#_Toc128906384)

[Table S21. Coding of outcome variables 27](#_Toc128906385)

[Table S22. Sensitivity Analyses: Cause-specific model results including partially and fully adjusted results for cohorts with mortality data 28](#_Toc128906386)

[Table S23. Subgroup analyses comparing partially adjusted models for Asian versus Western cohorts 29](#_Toc128906387)

[Figure S1. Funnel plot for Mild Cognitive Impairment – Fully-adjusted models 30](#_Toc128906388)

[Figure S2. Funnel plot for Dementia – Fully-adjusted models 31](#_Toc128906389)

[Figure S3. Funnel plot for Mortality– Fully-adjusted models 32](#_Toc128906390)

# Table S1. STROBE statement checklist for cohort studies

|  | **Item No** | **Recommendation** |
| --- | --- | --- |
| **Title and abstract** | 1 | (*a*) Indicate the study’s design with a commonly used term in the title or the abstract |
|  |  | Title |
|  |  | (*b*) Provide in the abstract an informative and balanced summary of what was done and what was found  Abstract |
| **Introduction** | | |
| Background/rationale | 2 | Explain the scientific background and rationale for the investigation being reported  Introduction: paragraphs 1-6 |
| Objectives | 3 | State specific objectives, including any prespecified hypotheses  Introduction, paragraphs 7-8 |
| **Methods** | | |
| Study design | 4 | Present key elements of study design early in the paper  Methods |
| Setting | 5 | Describe the setting, locations, and relevant dates, including periods of recruitment, exposure, follow-up, and data collection  Methods |
| Participants | 6 | (*a*) Give the eligibility criteria, and the sources and methods of selection of participants. Describe methods of follow-up  Methods: Contributing studies and participants, section.  Table 1. |
|  |  | (*b*) For matched studies, give matching criteria and number of exposed and unexposed  NA |
| Variables | 7 | Clearly define all outcomes, exposures, predictors, potential confounders, and effect modifiers. Give diagnostic criteria, if applicable  Methods: Measures section.  S3-S10, S12-S16, S20 Tables.  Methods: Statistical Analysis section. |
| Data sources/ measurement | 8* | For each variable of interest, give sources of data and details of methods of assessment (measurement). Describe comparability of assessment methods if there is more than one group  Methods: Measures section.  Tables S3-S10, S12-S16, S21 |
| Bias | 9 | Describe any efforts to address potential sources of bias  Methods: Sample section. |
| Study size | 10 | Explain how the study size was arrived at  Results: Sample section  Table 1 |
| Quantitative variables | 11 | Explain how quantitative variables were handled in the analyses. If applicable, describe which groupings were chosen and why  Methods: Statistical Analyses section  Tables S3-S10, S12-S16, S21 |
| Statistical methods | 12 | (*a*) Describe all statistical methods, including those used to control for confounding  Methods: Statistical Analyses section. |
|  |  | (*b*) Describe any methods used to examine subgroups and interactions  Methods: Statistical Analyses section |
|  |  | (*c*) Explain how missing data were addressed  Methods: Statistical Analyses- Missing data section |
|  |  | (*d*) If applicable, explain how loss to follow-up was addressed  Methods: Statistical Analyses- Two stage meta-analyses section |
|  |  | (*e*) Describe any sensitivity analyses  Methods: Statistical Analyses- Additional analyses section |
| **Results** | | |
| Participants | 13* | *(a)* Report numbers of individuals at each stage of study—eg numbers potentially eligible, examined for eligibility, confirmed eligible, included in the study, completing follow-up, and analysed  Tables 1 |
|  |  | *(b)* Give reasons for non-participation at each stage  With 13 different contributing studies and varying numbers of assessment waves, this level of information is not appropriate here, though can be found via the references for the studies listed in Table 2. |
|  |  | *(c)* Consider use of a flow diagram  Thirteen different contributing studies and varying numbers of assessment waves preclude the use of a manageable flow diagram. |
| Descriptive data | 14* | *(a)* Give characteristics of study participants (eg demographic, clinical, social) and information on exposures and potential confounders  Table 1  Tables S11, S17, S18 |
|  |  | *(b)* Indicate number of participants with missing data for each variable of interest  Tables S11, S17-S19 |
|  |  | *(c)* Summarise follow-up time (eg, average and total amount)  Table 1 |
| Outcome data | 15* | Report numbers of outcome events or summary measures over time  Table 2  Table S20 |
| Main results | 16 | *(a)* Give unadjusted estimates and, if applicable, confounder-adjusted estimates and their precision (eg, 95% confidence interval). Make clear which confounders were adjusted for and why they were included  Methods: Statistical Analysis section  Figures 1-3  Table 3  Table S22-S23 |
|  |  | *(b)* Report category boundaries when continuous variables were categorized  Methods: Measures section.  Tables S3-S10, S12-S16 |
|  |  | *(c)* If relevant, consider translating estimates of relative risk into absolute risk for a meaningful time period  NA |
| Other analyses | 17 | Report other analyses done—eg analyses of subgroups and interactions, and sensitivity analyses  Table S22-S23, Figure 4 |
| **Discussion** | | |
| Key results | 18 | Summarise key results with reference to study objectives  Discussion- paragraphs 1-4 |
| Limitations | 19 | Discuss limitations of the study, taking into account sources of potential bias or imprecision. Discuss both direction and magnitude of any potential bias  Discussion: Limitations section |
| Interpretation | 20 | Give a cautious overall interpretation of results considering objectives, limitations, multiplicity of analyses, results from similar studies, and other relevant evidence  Conclusion section |
| Generalisability | 21 | Discuss the generalisability (external validity) of the study results  Discussion: Strengths section |
| **Other information** | | |
| Funding | 22 | Give the source of funding and the role of the funders for the present study and, if applicable, for the original study on which the present article is based  Funding section |

# Table S2. Ethics approvals for each cohort study contributing to the current study’s dataset.

| **Study** | **Ethics approval** |
| --- | --- |
| Bambui Cohort Study of Ageing (BAMBUI) | Ethics Boards of the Fundac¸a˜o Oswaldo Cruz in Rio de Janeiro and the Instituto Rene´ Rachou of the Fundac¸a˜o Oswaldo Cruz in Belo Horizonte, Brazil (14/2007 - CEPSH-CpqRR) |
| Chinese Longitudinal Study of Ageing (CLAS) | Ethics Committee of Shanghai Mental Health Center (2011-YJ-14) |
| English Longitudinal Study of Ageing (ELSA) | The National Research and Ethics Committee granted Ethical approval for all the ELSA waves (MREC/01/2/91) |
| Epidemiology of Dementia in Central Africa (EPIDEMCA) | Ethical committees, supervised by Ministry of Public Health in Central African Republic ((8/UB/FACSS/CSCVPER/11)), the CERSSA (Comité d’Ethique de la Recherche en Sciences de Santé - 00000204) in the Republic of Congo, approved the study protocol, as well as the CPP-SOOM-IV (Comité de la Protection des Personnes Sud-Ouest Outre-Mer) in France. |
| Gothenberg H70 Birth Cohort Studies (the H70 study) | Regional Ethical Review Board (No. 075-09) |
| Hellenic Longitudinal Investigation of Ageing & Diet (HELIAD) | Institutional Ethics Review Board of the University of Thessaly (ΒΕΥ846Ψ8Ν2-32Π) and of Aiginition Hospital (ΑΔΑ: ΒΕΥ846Ψ8Ν2-32Π) |
| Korean Longitudinal Study on Cognitive Aging and Dementia (KLOSCAD) | Institutional Review Board of Seoul National University Bundang Hospital, Korea (IRB No. B-0912/089-010) |
| Leipzig Longitudinal Study of the Aged (LEILA75+) | Ethics committee of the University of Leipzig (C7 79934700) |
| Neuroprotective Model for Healthy Longevity among Malaysian Older Adults Towards Using Ageing (LRGS TUA) | National University of Malaysia (UKM) research ethics committee (reference: UKM PPI/111/8/JEP-2019-024), and was also approved by the Malaysian National Medical Research and Ethics Committee at the Malaysian Ministry of Health [reference: KKM/NIHSEC/P19-1689(12)]. |
| Sydney Memory and Ageing Study (MAS) | University of New South Wales Human Research Ethics Committee (approval #14327) |
| Monongahela-Youghiogheny Healthy Aging Team (MYHAT) | University of Pittsburgh Human Research Protection Office (formerly Institutional Review Board, IRB). Approval # PRO16030244 |
| Puerto Rican Elderly Health Conditions Study (PREHCO) | UAB IRB-160318001 |
| Singapore Longitudinal Study of Ageing (SLAS) | National University of Singapore Institutional Review Board (Reference Code: 04-140) |

Written consent was exclusively or predominantly obtained from participants in all studies. For EPIDEMCA, consent was obtained from family when the subject was unable to express his/her consent. For both EPIDEMCA and LRGS TUA, verbal consent and thumbprint mark was obtained from illiterate people. Further participant consent was not deemed necessary as only fully de-identified data were shared with the analysis team (e.g., as per the Privacy Rule proposed by the National Institute of Health, USA: http://privacyruleandresearch.nih.gov/research_repositories.asp).

# Table S3. Relationship Status Harmonisation

| **Study** | **Single or never married = 0; Widowed = 1; Separated/divorced = 2; Married or in a relationshi*p =* 3** |
| --- | --- |
| BAMBUI | Single = 0, widowed = 1, divorced = 2, married = 3 |
| CLAS | N/A |
| ELSA | Single = 0, widowed = 1, legally separated/divorced = 2, married (first marriage)/remarried = 3 |
| EPIDEMCA | Never married = 0, widowed = 1, divorced/separated = 2, married/in relationshi*p =* 3 |
| The H70 study | Not married = 0, widowed = 1, divorced = 2, married man/married woman = 3 |
| HELIAD | Not married = 0, widowed = 1, divorced = 2, married = 3 |
| KLOSCAD | Widowed = 1, divorced = 2, married = 3. Note there are no single options here. |
| LEILA75+ | Single = 0, widowed = 1, divorced = 2, married or living with spouse or significant other = 3 |
| LRGSTUA | Single = 0, widowed = 1, divorced/separated, married = 3 |
| MAS | Never married = 0, widowed = 1, divorced/separated = 2, married/de facto = 3 |
| MYHAT | Single/never married = 0, widowed = 1, divorced/separated = 2, living as married/married = 3 |
| PREHCO | Never married = 0, widowed = 1, divorced/separated = 2, married/cohabitation = 3 |
| SLAS | Single = 0, widowed = 1, divorced/separated = 2, married = 3 |

N/A: not available

# Table S4. Living Situation Harmonisation

| **Study** | **Lives alone = 0; Lives with at least one person = 1** |
| --- | --- |
| BAMBUI | Total number of people living in the household (includes all): 1 = 0, 2 or more = 1 |
| CLAS | ‘Over the past past, you …’ stay away from family and live alone = 0; often move the residence and most of time live together with strangers/live together with students, colleagues or friends/live together with family = 1 |
| ELSA | Number of people in the household/computed: 1 = 0, 2 or more = 1. |
| EPIDEMCA | Number of persons in the household (participant included): 1 = 0, 2 or more = 1 |
| The H70 study | Cohabitant: no or not cohabitant = 0, yes, cohabitant = 1 |
| HELIAD | Number of persons living with the participant: 0 = 0, 1 or more = 1 |
| KLOSCAD | Cohabitants: living alone = 0, with spouse/with other family members/with spouse and other family members = 1 |
| LEILA75+ | Presence of cohabitants - Baseline: lives alone = 0, lives with spouse or significant other/lives with other family members or lives with others but not family members = 1 |
| MAS | Number of people lived with: 0 = 0, 2 or more = 1 |
| MYHAT | Number of cohabitants: 0 = 0, 2 or more = 1 |
| PREHCO | Total number of people in the household: 1 = 0, 2 or more = 1 |
| SLAS | ‘Who do you live with?’: alone = 0, others = 1 |

# Table S5. Social Interactions with Friends/Family Harmonisation

| **Study** | **Never = 0; Few times a year = 1; Few times a month = 2; One or more times per week = 3** |
| --- | --- |
| BAMBUI | ‘In the past month, how often have you visited or received visits from your children who do not live with you?’ or ‘Not considering your children, have you visited or received regular visits from relatives in the last month who do not live in the same house as you?’ or ‘How often do you find someone from your job social gatherings outside of work hours? (excludes lunches or snacks with coworkers on weekdays)’ or ‘Do you have friends, acquaintances or neighbors that visit you or that you visit? (not necessarily the same person each time)?’: Never or almost never = 0, less than once a month/once or twice a year: 1, one to three times a month = 2, daily/one to four times a week = 3 |
| CLAS | N/A |
| ELSA | ‘How often the respondent meets up with their children on average’ or ‘How often the respondent meets up with their relatives on average’ or ‘How often the respondent meets up with their friends on average’: less than once a year or never = 0, every few months/once or twice a year = 1, once or twice a month = 2, three or more times a week/once or twice a week = 3 |
| EPIDEMCA | N/A |
| The H70 study | ‘Contact with children, visits’ or ‘contact children telephone/email’ or  ‘contact with grandchildren, visits’ or ‘visiting/meeting other people’ or ‘contact with other people telephone/email’: never = 0, less than every three months = 1, at least one time per month/at least one time per month to one time every three weeks = 2, daily/at least one time per week = 3 |
| HELIAD | Out to visit friends or relatives: once a year or rarer = 0, many times a year (< once a month) = 1, many times a month (1-5 times a month) = 2, many times a week/everyday or almost everyday = 3 |
| KLOSCAD | Social activities (counts/month): 0 = 0, 1-4 =2, 5 or more = 3 |
| LEILA75+ | ‘How often do you see any of your children or other relatives to speak to?’ or ‘If you have friends in this community/neighbourhood, how often do you have a chat or do something with one of your friends?’ or ‘How often do you see any of your neighbours to have a chat with or do something with one of your friends?’: never/no relatives = 0, less often = 1, at least monthly = 2, daily/2-3 times a weekly/at least weekly = 3 |
| LRGSTUA | N/A |
| MAS | Face to face contacts per month: 0 = 0, 1 to 3 = 2, 4 or more = 3 |
| MYHAT | How often do you see family or friends who do not live in the same house as you?: very rarely, if ever = 0, once every two or three weeks/once a month = 2, three or more times a week/once or twice a week = 1 |
| PREHCO | N/A |
| SLAS | ‘Visits by children/relatives/friends’ or ‘phone calls by children/relatives/friends’: none = 0, at least once a year = 1, at least once a month = 2, at least once a week = 3. |

N/A: not available

# Table S6. Community Group Engagement Harmonisation

| **Study** | **Never = 0; A few times a year = 1; At least monthly = 2; At least weekly = 3** |
| --- | --- |
| BAMBUI | ‘How often do you go to church or religious services?’: Never or almost never / does not apply = 0, less than once a month/once or twice a year = 1, one to three times a month = 2, almost every day, more than four times a week / one to four times a week = 3 |
| CLAS | N/A |
| ELSA | N/A |
| EPIDEMCA | N/A |
| The H70 study | Attends meetings of retirement clubs or other clubs: never = 0, less than once every three months/at least once a month to once every three months = 1, at least once a month = 2, daily/at least once a week = 3 |
| HELIAD | Participates in either volunteer work, cards/games/bingo, classes, church, groups: many times a year/once a year or rarer = 1, many times a month = 2, every day or almost every day/many times a week = 3. Note there are no options for never. |
| KLOSCAD | N/A |
| LEILA75+ | N/A |
| LRGSTUA | N/A |
| MAS | ‘charity or volunteer work’ or ‘bowls frequency unit’ or ‘tennis frequency unit’: not at all = 0, once a year/several times a year = 1, several times a month = 2, everyday = 3 |
| MYHAT | How often do you attend meetings or activities related to churches, lodges, societies, volunteer groups, etc. put together?: never = 0, less than monthly = 1, monthly/more than monthly = 2, more than weekly,less than daily/daily = 3 |
| PREHCO | ‘How often do you participate in social activities organized by your church?’: never = 0, sometimes = 2, frequently = 3 |
| SLAS | ‘Attend church/temple/mosque’ or ‘play cards/games/bingos/mahjong’ or ‘senior citizen club activities’ or ‘social group activities’ or ‘play mahjong’ or ‘unpaid community work’: never or less than once a month = 0, once or more per month but less than once a week = 2, once or more per week = 3 |

N/A: not available

# Table S7. Degree of Support Harmonisation

| **Study** | **None = 0; Some = 1; Significant = 2** |
| --- | --- |
| BAMBUI | N/A |
| CLAS | Social Support Rating Scale item 5: Obtain support and help from family members (scores 1-20; used tertiles; 1-9; 10-12; 13-20) |
| ELSA | ‘How much respondent can rely on spouse/partner if they have a serious problem?’ or ‘How much the respondent can rely on their children if they have a problem?’: not at all = 0, a little/some = 1, a lot = 2 |
| EPIDEMCA | N/A |
| The H70 study | N/A |
| HELIAD | N/A |
| KLOSCAD | Perceived and objective Support received from people: none or usually none = 0, half = 1, usually or yes = 2 |
| LEILA75+ | N/A |
| LRGSTUA | N/A |
| MAS | N/A |
| MYHAT | ‘If you were ever (or are) sick or disabled, would there be at least one person to take care of you?’: no help at all (except possible emergency help) would be available / would have to pay someone to hel*p =* 0, help would only be available now and then = 1, at least one person would take care of you indefinitely / at least one person could and would take care of you for a short time = 2 |
| PREHCO | N/A |
| SLAS | ‘Is someone able to help you if you needed/wanted’: not at all or very little = 0, to some extent = 1, to a great extent = 2 |

N/A: not available

# Table S8. Someone to Confide In/Emotional Support Harmonisation

| **Study** | **No = 0; Yes = 1** |
| --- | --- |
| BAMBUI | N/A |
| CLAS | N/A |
| ELSA | ‘How much respondent can open up to their spouse/partner if they need to talk? or ‘How much respondent can open up to their children if they need to talk?’: a little/not at all = 0, a lot/some = 1 |
| EPIDEMCA | N/A |
| The H70 study | “Do you have more than one confidant to talk to?”: yes = 1, no = 0. |
| HELIAD | N/A |
| KLOSCAD | ‘Availability of someone to confide in’: none/usually none = 0, half/usually yes = 1 |
| LEILA75+ | N/A |
| LRGSTUA | N/A |
| MAS | N/A |
| MYHAT | ‘Do you feel close enough to any family or friends that you could confide in the about any difficulties or concerns?’: no = 0, yes = 1 |
| PREHCO | ‘Is there someone that helps you by visiting you, keeping you company or listening to your problems?’ no = 0, yes = 1 |
| SLAS | ‘Do you have someone to confide in?’: no = 0, yes = 1 |

N/A: not available

# Table S9. Relationship Satisfaction Harmonisation

| **Study** | **Very dissatisfied = 0; Dissatisfied = 1; Satisfied = 2; Very satisfied = 3** |
| --- | --- |
| BAMBUI | ‘Considering all things, how do you feel about your personal relationships?’: very dissatisfied = 0, dissatisfied/indifferent = 1, satisfied = 2, very satisfied = 3 |
| CLAS | N/A |
| ELSA | N/A |
| EPIDEMCA | N/A |
| The H70 study | 7-point likert scale ‘satisfied with social network’ (1 (not satisfied) to 7 (satisfied)): 1 = 0, 2/3/4 = 1, 5/6 = 2, 7 = 3. |
| HELIAD | N/A |
| KLOSCAD | N/A |
| LEILA75+ | ‘Satisfaction with Life - family life/children - Baseline’ or ‘Satisfaction with Life – friends/acquaintances - Baseline’: not satisfied at all = 0, not satisfied/neither satisfied nor dissatisfied = 1, satisfied = 2, absolutely satisfied = 3 |
| LRGSTUA | N/A |
| MAS | N/A |
| MYHAT | ‘How satisfied are you with the support and encouragement you receive from the people close to you?’: very dissatisfied = 0, somewhat dissatisfied = 1, somewhat satisfied = 2, very satisfied = 3. |
| PREHCO | N/A |
| SLAS | N/A |

N/A: not available

# Table S10. Loneliness Frequency (reverse scored) Harmonisation

| **Study** | **Never = 2; Sometimes = 1; Often = 0 (reverse scored)** |
| --- | --- |
| BAMBUI | N/A |
| CLAS | N/A |
| ELSA | How often respondent feels lonely: hardly ever or never = 2, some of the time = 1, often = 0 |
| EPIDEMCA | N/A |
| The H70 study | Feeling lonely: never = 2, seldom/sometimes= 1, often = 0 |
| HELIAD | N/A |
| KLOSCAD | N/A |
| LEILA75+ | CES-D - Item 14 – “I felt lonely”: rarely or none of the time (less than 1 day) = 2, some or a little, 1-2 days = 1, occasionally or a moderate amount, 3-4 days/most or all the time, 5-7 days = 0 |
| LRGSTUA | UCLA 3 item loneliness scale (Hughes et al. 2004) item 1 “How often do you feel that you lack companionship?”: hardly ever = 2, some of the time = 1, often = 0 |
| MAS | N/A |
| MYHAT | N/A |
| PREHCO | N/A |
| SLAS | N/A |

N/A: not available

# Table S11. Characteristics of Sample- Social Connections

|  | BAMBUI (N=1602) | CLAS (N=3059) | ELSA (N=9300) | EPIDEMCA (N=2001) | GothenburgH70 (N=1221) | HELIAD (N=2032) | KLOSCAD (N=6749) | LEILA75+ (N=1263) | LRGSTUA (N=2322) | MYHAT (N=1919) | PREHCO (N=3962) | SLAS (N=2804) | MAS (N=1037) | Total (N=39271) |
| --- | --- | --- | --- | --- | --- | --- | --- | --- | --- | --- | --- | --- | --- | --- |
| **Relationship Status** |  |  |  |  |  |  |  |  |  |  |  |  |  |  |
| Single or never married | 147 (9.7%) | 0 | 603 (7.5%) | 48 (2.4%) | 82 (8.0%) | 29 (1.5%) | 30 (0.5%) | 105 (8.3%) | 41 (1.8%) | 82 (4.3%) | 267 (6.7%) | 169 (6.0%) | 132 (12.8%) | 1735 (5.0%) |
| Widowed | 538 (35.6%) | 0 | 1061 (13.1%) | 1085 (54.4%) | 321 (31.2%) | 493 (25.4%) | 1761 (26.6%) | 706 (55.9%) | 655 (28.2%) | 753 (39.3%) | 1422 (35.9%) | 477 (17.0%) | 350 (33.8%) | 9622 (27.9%) |
| Separated or divorced | 79 (5.2%) | 0 | 1137 (14.1%) | 133 (6.7%) | 149 (14.5%) | 53 (2.7%) | 181 (2.7%) | 107 (8.5%) | 41 (1.8%) | 133 (6.9%) | 701 (17.7%) | 90 (3.2%) | 124 (12.0%) | 2928 (8.5%) |
| Married or in a relationship | 747 (49.4%) | 0 | 5268 (65.3%) | 730 (36.6%) | 478 (46.4%) | 1368 (70.4%) | 4648 (70.2%) | 345 (27.3%) | 1585 (68.3%) | 950 (49.5%) | 1572 (39.7%) | 2063 (73.7%) | 428 (41.4%) | 20182 (58.6%) |
| Missing (n) | 91 | 3059 | 1231 | 5 | 191 | 89 | 129 | 0 | 0 | 1 | 0 | 5 | 3 | 4804 |
| **Living Situation** |  |  |  |  |  |  |  |  |  |  |  |  |  |  |
| Living alone | 254 (16.0%) | 205 (6.9%) | 2024 (21.8%) | 26 (4.7%) | 714 (92.1%) | 361 (17.9%) | 983 (14.9%) | 635 (60.2%) | 246 (10.6%) | 745 (38.9%) | 1252 (31.6%) | 224 (8.0%) | 3 (0.3%) | 7672 (20.8%) |
| Not living alone | 1335 (84.0%) | 2762 (93.1%) | 7272 (78.2%) | 527 (95.3%) | 61 (7.9%) | 1653 (82.1%) | 5619 (85.1%) | 420 (39.8%) | 2076 (89.4%) | 1171 (61.1%) | 2710 (68.4%) | 2580 (92.0%) | 1034 (99.7%) | 29220 (79.2%) |
| Missing (n) | 13 | 92 | 4 | 1448 | 446 | 18 | 147 | 208 | 0 | 3 | 0 | 0 | 0 | 2379 |
| **Community Group Frequency** |  |  |  |  |  |  |  |  |  |  |  |  |  |  |
| Never | 238 (15.1%) | 0 | 0 | 0 | 22 (4.0%) | 0 (0.0%) | 0 | 0 | 0 | 71 (4.3%) | 1548 (45.0%) | 616 (22.3%) | 426 (43.2%) | 2921 (22.6%) |
| Few times a year | 277 (17.5%) | 0 | 0 | 0 | 202 (36.5%) | 590 (29.8%) | 0 | 0 | 0 | 121 (7.4%) | 0 (0.0%) | 0 (0.0%) | 200 (20.3%) | 1390 (10.7%) |
| At least monthly | 422 (26.7%) | 0 | 0 | 0 | 194 (35.0%) | 833 (42.1%) | 0 | 0 | 0 | 1009 (61.4%) | 1359 (39.5%) | 1340 (48.4%) | 197 (20.0%) | 5354 (41.3%) |
| At least weekly | 644 (40.7%) | 0 | 0 | 0 | 136 (24.5%) | 554 (28.0%) | 0 | 0 | 0 | 443 (26.9%) | 532 (15.5%) | 812 (29.3%) | 164 (16.6%) | 3285 (25.4%) |
| Missing (n) | 21 | 3059 | 9300 | 2001 | 667 | 55 | 6749 | 1263 | 2322 | 275 | 523 | 36 | 50 | 26321 |
| **Social Interactions Frequency** |  |  |  |  |  |  |  |  |  |  |  |  |  |  |
| Never | 20 (1.2%) | 0 | 53 (0.7%) | 268 (14.0%) | 1 (0.1%) | 576 (29.2%) | 2139 (34.9%) | 7 (0.6%) | 0 | 62 (3.2%) | 0 | 77 (2.8%) | 0 | 3203 (11.7%) |
| Few times a year | 36 (2.2%) | 0 | 511 (6.4%) | 176 (9.2%) | 0 (0.0%) | 486 (24.6%) | 0 (0.0%) | 31 (2.7%) | 0 | 0 (0.0%) | 0 | 66 (2.4%) | 0 | 1336 (4.9%) |
| Few times a month | 166 (10.4%) | 0 | 1380 (17.4%) | 120 (6.3%) | 23 (2.5%) | 667 (33.8%) | 1120 (18.3%) | 72 (6.3%) | 0 | 329 (17.2%) | 0 | 265 (9.6%) | 0 | 4252 (15.6%) |
| Once or more per week | 1378 (86.1%) | 0 | 6001 (75.5%) | 1348 (70.5%) | 885 (97.4%) | 245 (12.4%) | 2874 (46.9%) | 1029 (90.3%) | 0 | 1524 (79.6%) | 0 | 2360 (85.3%) | 0 | 18504 (67.8%) |
| Missing (n) | 2 | 3059 | 1355 | 89 | 312 | 58 | 616 | 124 | 2322 | 4 | 3962 | 36 | 1037 | 11976 |
| **Support Degree** |  |  |  |  |  |  |  |  |  |  |  |  |  |  |
| None | 0 | 907 (30.6%) | 5 (0.1%) | 0 | 0 | 0 | 818 (12.9%) | 0 | 0 | 212 (11.3%) | 0 | 233 (8.4%) | 0 | 2175 (10.2%) |
| Some | 0 | 984 (33.2%) | 601 (8.1%) | 0 | 0 | 0 | 1132 (17.8%) | 0 | 0 | 64 (3.4%) | 0 | 1271 (45.7%) | 0 | 4052 (18.9%) |
| Significant | 0 | 1069 (36.1%) | 6833 (91.9%) | 0 | 0 | 0 | 4409 (69.3%) | 0 | 0 | 1605 (85.3%) | 0 | 1280 (46.0%) | 0 | 15196 (70.9%) |
| Missing (n) | 1602 | 99 | 1861 | 2001 | 1221 | 2032 | 390 | 1263 | 2322 | 38 | 3962 | 20 | 1037 | 17848 |
| **Someone to confide in** |  |  |  |  |  |  |  |  |  |  |  |  |  |  |
| No | 0 | 0 | 170 (2.4%) | 0 | 318 (36.4%) | 0 | 1239 (19.5%) | 0 | 0 | 51 (2.7%) | 2299 (62.1%) | 142 (5.1%) | 51 (5.2%) | 4270 (18.0%) |
| Yes | 0 | 0 | 7004 (97.6%) | 0 | 555 (63.6%) | 0 | 5120 (80.5%) | 0 | 0 | 1864 (97.3%) | 1405 (37.9%) | 2639 (94.9%) | 928 (94.8%) | 19515 (82.0%) |
| Missing (n) | 1602 | 3059 | 2126 | 2001 | 348 | 2032 | 390 | 1263 | 2322 | 4 | 258 | 23 | 58 | 15486 |
| **Relationship Satisfaction** |  |  |  |  |  |  |  |  |  |  |  |  |  |  |
| Very Dissatisfied | 26 (1.6%) | 0 | 0 | 0 | 5 (0.7%) | 0 | 0 | 2 (0.2%) | 0 | 22 (1.2%) | 0 | 0 | 0 | 55 (1.0%) |
| Dissatisfied | 164 (10.3%) | 0 | 0 | 0 | 54 (7.1%) | 0 | 0 | 62 (5.6%) | 0 | 24 (1.3%) | 0 | 0 | 0 | 304 (5.7%) |
| Satisfied | 1208 (76.1%) | 0 | 0 | 0 | 226 (29.7%) | 0 | 0 | 690 (61.8%) | 0 | 143 (7.7%) | 0 | 0 | 0 | 2267 (42.5%) |
| Very Satisfied | 189 (11.9%) | 0 | 0 | 0 | 477 (62.6%) | 0 | 0 | 362 (32.4%) | 0 | 1675 (89.9%) | 0 | 0 | 0 | 2703 (50.7%) |
| Missing (n) | 15 | 3059 | 9300 | 2001 | 459 | 2032 | 6749 | 147 | 2322 | 55 | 3962 | 2804 | 1037 | 33942 |
| **Loneliness** |  |  |  |  |  |  |  |  |  |  |  |  |  |  |
| Often | 0 | 0 | 438 (5.5%) | 0 | 44 (4.9%) | 0 | 0 | 176 (17.0%) | 34 (1.5%) | 0 | 0 | 0 | 0 | 692 (5.7%) |
| Rarely or Sometimes | 0 | 0 | 1821 (23.0%) | 0 | 488 (53.9%) | 0 | 0 | 249 (24.1%) | 190 (8.4%) | 0 | 0 | 0 | 0 | 2748 (22.7%) |
| Never | 0 | 0 | 5654 (71.5%) | 0 | 373 (41.2%) | 0 | 0 | 609 (58.9%) | 2045 (90.1%) | 0 | 0 | 0 | 0 | 8681 (71.6%) |
| Missing (n) | 1602 | 3059 | 1387 | 2001 | 316 | 2032 | 6749 | 229 | 53 | 1919 | 3962 | 2804 | 1037 | 27150 |

# Table S12. Smoking Harmonisation

| **Study** | **Current: 0 (no), 1 (yes)** |
| --- | --- |
| BAMBUI | Self-reported current smoker |
| CLAS | 1 or more cigarettes per day |
| ELSA | Whether smokes cigarettes at all nowadays |
| EPIDEMCA | N/A |
| The H70 study | Current cigarette smoker |
| HELIAD | Self-reported current smoking |
| KLOSCAD | Current smoking pack/day 1+ |
| LEILA75+ | 1 or more cigarettes per day |
| LRGSTUA | Whether or not participant currently smokes |
| MAS | 1 or more cigarette per day |
| MYHAT | Whether participant currently smokes |
| PREHCO | Whether or not participant currently smokes |
| SLAS | Frequency of smoking = never or less than once a month |

N/A: not available

# Table S13. Diabetes Mellitus Harmonisation

| **Study** | **Current: 0 (no), 1 (yes - meeting any is sufficient)** |
| --- | --- |
| BAMBUI | 1. Fasting blood glucose, 2. Treatment |
| CLAS | Self-reported history of diabetes |
| ELSA | 1.Has been told they have diabetes, 2. Treatment for diabetes |
| EPIDEMCA | 1. Has been told they have diabetes, 2. Treatment for diabetes |
| The H70 study | Self-reported history of diabetes |
| HELIAD | Self-reported history of diabetes |
| KLOSCAD | 1. Current status of diabetes, 2. Fasting glucose levels |
| LEILA75+ | Self-reported diabetes or treatment |
| LRGSTUA | 1. Self-reported diabetes, 2. Self-reported diabetes diagnosed by a doctor |
| MAS | 1. Fasting blood glucose, 2. Treatment for diabetes, 3. History of diabetes |
| MYHAT | Self-reported diabetes |
| PREHCO | Self-reported diabetes or treatment |
| SLAS | 1. Fasting blood glucose, 2. Treatment for diabetes, 3. History of diabetes |

Fasting blood glucose criteria are ≥126mg/dL or >7mmol/L. N/A: not available

# Table S14. Hypertension Harmonisation

| **Study** | **Current: 0 (no), 1 (yes - meeting any is sufficient)^a^** |
| --- | --- |
| BAMBUI | 1. Previous medical diagnosis of hypertension, 2. Blood pressure measurements |
| CLAS | 1. Self-reported hypertension, 2. Treatment for hypertension |
| ELSA | Blood pressure measurements |
| EPIDEMCA | 1. Self-reported hypertension with treatment, 2. Blood pressure measurements |
| The H70 study | 1. Self-reported hypertension with treatment, 2. Blood pressure measurements |
| HELIAD | Self-reported hypertension |
| KLOSCAD | 1. Self-reported hypertension current, 2. History of hypertension |
| LEILA75+ | Proxy report of hypertension |
| LRGSTUA | 1. Self-reported diagnosis of high blood pressure by doctor, 2. Self-reported high blood pressure |
| MAS | High blood pressure diagnosed by doctor |
| MYHAT | 1. Self-reported hypertension, 2. Blood pressure measurements |
| PREHCO | Self-reported diagnosis of high blood pressure by doctor |
| SLAS | N/A |

**^a^**  Blood pressure criteria are seated systolic blood pressure ≥140 mmHg or diastolic blood pressure ≥90 mmHg. N/A: not available

# Table S15. Cardiovascular Disease Harmonisation

| **Study** | **Current: 0 (no), 1 (yes - meeting any is sufficient)** |
| --- | --- |
| BAMBUI | Self-reported history of angina or myocardial infarction |
| CLAS | Self-reported history of heart disease, angina or myocardial infarction |
| ELSA | Self-reported history of angina, heart attack or congestive heart failure |
| EPIDEMCA | N/A |
| The H70 study | Self-reported myocardial infarction |
| HELIAD | Self-reported history of coronary disease, myocardial infarction |
| KLOSCAD | Self-reported history of angina or myocardial infarct |
| LEILA75+ | Self-reported heart failure |
| LRGSTUA | 1. Self-reported heart disease diagnosed by doctor, 2. Self-reported heart disease |
| MAS | Self-reported heart problems, heart attack, angina, atrial fibrillation or cardiac arrhythmia requiring treatment |
| MYHAT | Self-reported heart attack, congestive heart failure or cardiac arrest |
| PREHCO | 1. Self-reported angina, coronary disease or congestive heart disease, 2. Medication taken for heart condition |
| SLAS | Presence of cardiac disease |

# Table S16. Depression Harmonisation

| **Study** | **Current: 0 (no), 1 (yes - meeting any is sufficient)** |
| --- | --- |
| BAMBUI | General health questionnaire 12 score of 5+ |
| CLAS | Whether has depression at baseline |
| ELSA | Self-reported depression |
| EPIDEMCA | Self-reported depressive symptoms |
| The H70 study | Self-reported major or minor depression |
| HELIAD | Geriatric depression scale (GDS) Score 5+ |
| KLOSCAD | 1. Current status of depressive disorder under observation or under treatment, 2. Korean Geriatric Depression Scale 16+ |
| LEILA75+ | 1. CES-D score 16 +, 2. DSM-IV clinical interview |
| LRGSTUA | GDS score 6+ |
| MAS | GDS score 6+ |
| MYHAT | Modified CES-D score 3+ |
| PREHCO | N/A |
| SLAS | Use of anti-depressants |

# Table S17. Characteristics of Sample- Covariates

|  | **BAMBUI (N=1602)** | **CLAS (N=3059)** | **ELSA (N=9300)** | **EPIDEMCA (N=2001)** | **GothenburgH70 (N=1221)** | **HELIAD (N=2032)** | **KLOSCAD (N=6749)** | **LEILA75+ (N=1263)** | **LRGSTUA (N=2322)** | **MYHAT (N=1919)** | **PREHCO (N=3962)** | **SLAS (N=2804)** | **MAS (N=1037)** | **Total (N=39271)** |
| --- | --- | --- | --- | --- | --- | --- | --- | --- | --- | --- | --- | --- | --- | --- |
| **Current Smoking** |  |  |  |  |  |  |  |  |  |  |  |  |  |  |
| No | 1303 (81.3%) | 2167 (72.0%) | 4879 (82.0%) | 133 (23.3%) | 629 (86.3%) | 1750 (89.3%) | 5821 (88.7%) | 838 (85.4%) | 1923 (82.8%) | 1781 (93.1%) | 1039 (76.6%) | 2581 (93.2%) | 476 (46.0%) | 25320 (82.3%) |
| Yes | 299 (18.7%) | 841 (28.0%) | 1074 (18.0%) | 439 (76.7%) | 100 (13.7%) | 209 (10.7%) | 745 (11.3%) | 143 (14.6%) | 399 (17.2%) | 131 (6.9%) | 318 (23.4%) | 189 (6.8%) | 559 (54.0%) | 5446 (17.7%) |
| Missing (n) | 0 | 51 | 3347 | 1429 | 492 | 73 | 183 | 282 | 0 | 7 | 2605 | 34 | 2 | 8505 |
| **Diabetes** |  |  |  |  |  |  |  |  |  |  |  |  |  |  |
| No | 1206 (85.0%) | 2183 (81.9%) | 8381 (90.2%) | 1916 (96.0%) | 785 (90.9%) | 1010 (81.2%) | 3967 (71.0%) | 963 (77.1%) | 1717 (73.9%) | 1501 (78.3%) | 2859 (72.4%) | 2340 (83.5%) | 839 (83.7%) | 29667 (81.7%) |
| Yes | 213 (15.0%) | 484 (18.1%) | 915 (9.8%) | 79 (4.0%) | 79 (9.1%) | 234 (18.8%) | 1623 (29.0%) | 286 (22.9%) | 605 (26.1%) | 416 (21.7%) | 1091 (27.6%) | 464 (16.5%) | 163 (16.3%) | 6652 (18.3%) |
| Missing (n) | 183 | 392 | 4 | 6 | 357 | 788 | 1159 | 14 | 0 | 2 | 12 | 0 | 35 | 2952 |
| **Cardiovascular Disease** |  |  |  |  |  |  |  |  |  |  |  |  |  |  |
| No | 1282 (86.6%) | 2147 (73.5%) | 8863 (95.3%) | 0 | 801 (92.3%) | 1483 (75.9%) | 6098 (93.7%) | 1144 (92.1%) | 2083 (89.7%) | 1515 (79.0%) | 3204 (81.4%) | 2573 (91.8%) | 673 (64.9%) | 31866 (87.8%) |
| Yes | 198 (13.4%) | 774 (26.5%) | 436 (4.7%) | 0 | 67 (7.7%) | 471 (24.1%) | 408 (6.3%) | 98 (7.9%) | 239 (10.3%) | 402 (21.0%) | 734 (18.6%) | 231 (8.2%) | 364 (35.1%) | 4422 (12.2%) |
| Missing (n) | 122 | 138 | 1 | 2001 | 353 | 78 | 243 | 21 | 0 | 2 | 24 | 0 | 0 | 2983 |
| **Depression** |  |  |  |  |  |  |  |  |  |  |  |  |  |  |
| No | 926 (61.4%) | 2756 (93.0%) | 8601 (92.8%) | 1238 (61.9%) | 852 (83.7%) | 1667 (84.5%) | 4891 (76.8%) | 870 (68.9%) | 1114 (85.0%) | 1680 (87.8%) | 0 | 2751 (98.1%) | 957 (92.7%) | 28303 (84.7%) |
| Yes | 581 (38.6%) | 209 (7.0%) | 672 (7.2%) | 763 (38.1%) | 166 (16.3%) | 305 (15.5%) | 1479 (23.2%) | 393 (31.1%) | 197 (15.0%) | 233 (12.2%) | 0 | 53 (1.9%) | 75 (7.3%) | 5126 (15.3%) |
| Missing (n) | 95 | 94 | 27 | 0 | 203 | 60 | 379 | 0 | 1011 | 6 | 3962 | 0 | 5 | 5842 |
| **Hypertension** |  |  |  |  |  |  |  |  |  |  |  |  |  |  |
| No | 576 (38.6%) | 1529 (51.7%) | 4417 (68.8%) | 648 (32.4%) | 168 (16.2%) | 691 (35.2%) | 3044 (46.7%) | 528 (67.2%) | 1154 (49.7%) | 512 (26.7%) | 1648 (41.7%) | 0 | 404 (39.1%) | 15319 (47.3%) |
| Yes | 916 (61.4%) | 1430 (48.3%) | 2000 (31.2%) | 1350 (67.6%) | 868 (83.8%) | 1272 (64.8%) | 3479 (53.3%) | 258 (32.8%) | 1168 (50.3%) | 1406 (73.3%) | 2303 (58.3%) | 0 | 629 (60.9%) | 17079 (52.7%) |
| Missing (n) | 110 | 100 | 2883 | 3 | 185 | 69 | 226 | 477 | 0 | 1 | 11 | 2804 | 4 | 6873 |

# Table S18. Cognitive tests used to determine mild cognitive impairment and their descriptive statistics at baseline.

| Study | Global Cognition | Memory | Language | Executive functioning | Perceptual Motor | Attention/Processing Speed |
| --- | --- | --- | --- | --- | --- | --- |
| BAMBUI | MMSE Total (*M* = 24·3, *SD* = 4·8) | **~~-~~** | - | - | - | - |
| CLAS | MMSE Total (*M* = 25·1, *SD* = 5·5) | AVLT Trial (*M* = 7·1, *SD* = 4·5) | FAS equivalent Verbal Fluency (*M* = 26·1, *SD* = 11.0) | Digit Span Backward (*M* = 4.9, *SD* = 2·7) | Block Design (WAIS-III) (*M* = 24.2, *SD* = 10.6) | Digit Span Forward (*M* = 8.6, *SD* = 4.8) |
| ELSA | - | TICSm Memory scale: Word list recall (*M* = 4·6, *SD* = 2·2) | - | TICSm Executive scale score)- comprises of Serial 7s and Counting Backwards from 20 to 0 (*M* = 3·8, *SD* = 1·7) | - | - |
| EPIDEMCA | CSI-D (*M* = 25.6, *SD* = 5.3) | Free and Cued Selective Reminding Test (*M* = 8.0, *SD* = 3.9) | Animals in 60s (*M* = 7·6, *SD* = 3·1) | - | - | - |
| the H70 study | MMSE Total (*M* = 27·0, *SD* = 4·5) | MMSE recall (*M* = 1·58, *SD* = 1.0) | Animals in 60s (*M* = 20·5, *SD* = 7·7) | Digit Span Backward (*M* = 4·2, *SD* = 1·1) | Block Design (*M* = 19.7, *SD* = 7.0) | Digit Span Forward (*M =* 5.8, *SD* = 1.3) |
| HELIAD | MMSE Total (*M* = 26·7, *SD* = 3.4) | Greek Verbal Learning Test (*M* = 10·6, *SD* = 3·3) | Objects in 60s (*M* = 15.0, *SD* = 5·4) | Trail Making Test B* (*M* = -172·2, *SD* = 73·9) | Executive Clock Drawing Task (*M* = 13.8, *SD* = 1.7) | Trail Making Test A*  (*M* = 88.6, *SD* = 51.0) |
| KLOSCAD | MMSE Total (*M* = 25·2, *SD* = 4.3) | CERAD Word-list Recall Test (*M* = 4·5, *SD* = 2·2) | Animals in 60s (*M* = 13·5, *SD* = 4·7) | Trail Making Test B* (*M* =-168·6, *SD* = 72·2) | Executive Clock Drawing Task (*M* = 11.3, *SD =* 4.2) | Trail Making Test A* (*M* = 79.4, *SD* = 54.7) |
| LEILA75+ | MMSE Total (*M* = 24.5, *SD* = 6.4) | SIDAM memory score (*M* = 14.0, *SD* = 4.7) | - | SIDAM Intellectual abilities score (*M* = 4·1, *SD* = 1.4) | SIDAM Constructional Abilities Subtest score (M = 1.4, *SD* = 1.1) | - |
| LRGSTUA | MMSE Total (*M* = 22·8, *SD* = 4·9) | Rey Auditory Verbal Learning Test (*M* = 4·6, *SD* = 3·6) | - | Digit Symbol (*M* = 26·2, *SD* = 15·6) | - | - |
| MAS | MMSE Total (*M* = 28·0, *SD* = 1·5) | Rey Auditory Verbal Learning Test, trial 7 (*M* = 7·5, *SD* = 3·5) | Animals in 60s (*M* = 15·6, *SD* = 4·4) | Trail Making Test B* (*M* = -118·5, *SD* = 49·4) | Block Design (WAIS-R) (*M* = 21.3, *SD* = 8.2) | Trail Making Test A* (*M* = 47.0, *SD =* 16.6) |
| MYHAT | MMSE Total (*M* = 27.0, *SD* = 2·4) | WMS-R Logical Memory delayed recall (*M* = 14·5, *SD* = 7·6) | Animals in 60s (*M* = 15·6, *SD* = 4·9) | Trail Making Test B* (*M* = -118·8, *SD* = 51.0) | Block Design (*M* = 28.5, *SD* = 9.4) | Trail Making Test A* (*M* = 48.5, *SD* = 25.9) |
| PREHCO | MMC (*M* = 16·2, *SD* = 3·0) | MMSE recall (*M* = 2·0, *SD* = 1·1) | - | - | - | - |
| SLAS | MMSE Total (*M* = 26.9, *SD* = 3·7) | Rey Auditory Verbal Learning Test (*M* = 15.4, *SD* = 4.2) | Animals in 60s (*M* = 15·4, *SD* = 4·2) | Trail Making Test B* (*M* = -118·8, *SD* = 63·3) | Block Design (*M* = 22.8, *SD* = 10.5) | Trail Making Test A* (*M* = 53.7, *SD* = 29.9)­­­ |

**Note:** MMSE = Mini-mental state examination. CERAD = Consortium to Establish a Registry for Alzheimer Disease. CSI-D = Cognitive State Interview- Dementia. SIDAM = Structured Interview for the diagnosis of Dementia of the Alzheimer type, multi-infarct dementia and dementias of other etiology according to ICD-10 and DSM-IV. TICSm = Telephone Interview for Cognitive Status modified. WAIS-R = Wechsler Adult Intelligence Scale- Revised. WAIS-III = Wechsler Adult Intelligence Scale- Third Edition. MMC = Mini-Mental Cabán. *Spikes removed from data before analysis. Trail Making Test timings were reversed so that higher values indicated better performance.

# **Table S19. Percentage of missing data at baseline wave for global cognition and all cognitive domains.**

| **Study** | **Global Cognition (%)** | **Memory (%)** | **Language (%)** | **Executive Functioning (%)** | **Perceptual Motor (%)** | **Attention/Processing Speed (%)** |
| --- | --- | --- | --- | --- | --- | --- |
| BAMBUI | 3·12 | NA | NA | NA | NA | NA |
| CLAS | 0·918 | 4·07 | 3·44 | 2·03 | 3·48 | 2·97 |
| ELSA | NA | 5·22 | NA | 12·03 | NA | NA |
| EPIDEMCA | 0·05 | 85·485 | 79·16 | NA | NA | NA |
| The H70 study | 20·25 | 19·36 | 19·09 | 80·64 | 80·46 | 80·64 |
| HELIAD | 10·05 | 5·23 | 3·58 | 10·47 | 73·32 | 1·35 |
| KLOSCAD | 1·27 | 3·23 | 2·92 | 37·84 | 5·52 | 8·52 |
| LEILA75+ | 0·10 | 14·93 | NA | 14·93 | 15·22 | NA |
| LRGSTUA | 1·70 | 6·27 | NA | 11·45 | NA | NA |
| MAS | 0·00 | 1·06 | 0·48 | 8·68 | 0·29 | 1·54 |
| MYHAT | 0·00 | 3·06 | 2·37 | 6·33 | 9·02 | 2·74 |
| PREHCO | 9·14 | 9·14 | NA | NA | NA | NA |
| SLAS | 0·33 | 74·95 | 75·14 | 91·80 | 93·80 | 89·87 |

# Supplementary Text S1. Standardization of cognitive outcome scores

Neuropsychological tests were categorised as assessing global cognition, memory, language, or executive function based on previous studies^18^. Only cognitive tests with less than 20% missing data were retained (see missing data proportion in Table S4). In individuals without dementia, cognitive scores were standardized following previous protocol^19^. First, raw scores within each study, pooled across waves, were normalized using Blom's formula (i.e., transformed to a normal distribution). Transformed test scores with values outside plus or minus 3 SDs were winsorized to reduce the impact of outliers. These scores were then standardized in each study by converting to z-scores using the estimated baseline mean and SD in the individuals without dementia, at common values of age, sex, and education, obtained from pooling data across all studies.

# Table S20. Identification of people with dementia

| **Study** | **Dementia diagnosis** |
| --- | --- |
| BAMBUI | MMSE < 14 |
| CLAS | Consensus diagnosis |
| ELSA | Self-reported dementia diagnosis or average IQCODE ≥ 3·38 |
| EPIDEMCA | Consensus diagnosis |
| The H70 study | Consensus diagnosis |
| HELIAD | Consensus diagnosis |
| KLOSCAD | Consensus diagnosis |
| LEILA75+ | Consensus diagnosis |
| LRGSTUA | MMSE ≤ 2 SD and IADL < 6 or Clinical Dementia Rating ≥ 1, if available |
| MAS | Consensus diagnosis |
| MYHAT | Clinical Dementia Rating ≥ 1 |
| PREHCO | Mini-mental Caban ≤ 1.5 SD below mean |
| SLAS | Consensus diagnosis |

Note. In order to identify people with dementia, we used consensus diagnoses wherever available and used established cut-off scores for specific populations. Consensus diagnoses were available for most studies (except BAMBUI, PREHCO, MYHAT, and ELSA). For BAMBUI and PREHCO, published cut-off scores for probable cognitive impairment/dementia were used (the BAMBUI MMSE cut-off is 13/14^14^; the PREHCO cut-off is ≤ 1·5 SD below mean on the Mini-mental Caban^12,15^. For MYHAT, a Clinical Dementia Rating of 1 or greater was used to identify PLWD^16^. For ELSA, we used self-reported dementia diagnosis or an average IQCODE score of 3·38 or higher to identify PLWD^17^.

# Table S21. Coding of outcome variables

| **Model** | **Outcome** | **Coding** | **Excluded from model** | **Sample size for meta-analytic model** |
| --- | --- | --- | --- | --- |
| Cause specific hazard model | MCI | 0 = Censored or Death  1 = MCI/Dementia^a^ | People with MCI/dementia at baseline (*n* = 10, 328) | 28,915 |
| Cause specific hazard model | Dementia | 0 = Censored or Death  1 = Dementia | People with dementia at baseline (*n* = 1031) | 37,297 |
| Cox regression model | Mortality | 0 = Censored  1 = Death | - | 26,907 |
| Subdistribution hazard model | Mild cognitive impairment with competing risk for mortality | 0 = Censored  1 = MCI/Dementia^a^  2 = Death | People with MCI/dementia at baseline (*n* = 10328) | 18,149 |
| Subdistribution hazard model | Dementia with competing risk for mortality | 0 = Censored  1 = Dementia  2 = Death | People with dementia at baseline (*n* = 1031) | 25,473 |

^a^ Participants who were diagnosed with dementia are assumed to have progressed from MCI at some stage. This event denotes whichever was first.

#

# Table S22. Sensitivity Analyses: Cause-specific model results including partially and fully adjusted results for cohorts with mortality data

|  | **Social Connection Marker** | **Partially adjusted models** | | | | **Fully adjusted models** | | | |
| --- | --- | --- | --- | --- | --- | --- | --- | --- | --- |
|  |  | **csHR (95%CI)** | *I^2^* (%) | *τ^2^* | Egger’s test^b^ | **csHR (95%CI)** | *I^2^* (%) | *τ^2^* | Egger’s test |
| MCI | Married/in a relationship | 0.90 (0.74, 1.09) | 26.81 | 0.02 | *z =* 1.66, *p =* 0.10 | 0.87 (0.72, 1.06) | 19.00 | 0.01 | *z =* 2.02, *p =* 0.04 |
|  | Living with others | 0.90 (0.79, 1.03) | 49.29 | 0.02 | *z =* -1.03, *p =* 0.30 | 0.88 (0.76, 1.02) | 45.68 | 0.02 | *z =* -1.07, *p =* 0.29 |
|  | **Community Group Engagement^a^** |  |  |  |  |  |  |  |  |
|  | Yearly | 0.90 (0.73, 1.12) | 0.00 | 0.00 | *z =* 1.66, *p =* 0.10 | 0.86 (0.68, 1.09) | 0.01 | 0.00 | *z =* 1.48, *p =* 0.14 |
|  | Monthly | 1.00 (0.82, 1.23) | 8.68 | 0.01 | *z =* 0.42, *p =* 0.68 | 1.05 (0.81, 1.35) | 15.81 | 0.01 | *z =* 0.96, *p =* 0.34 |
|  | **Weekly** | 0.76 (0.56, 1.03) | 42.01 | 0.05 | *z =* 1.35, *p =* 0.18 | **0.70 (0.54, 0.92)** | 17.57 | 0.01 | *z =* 1.91, *p =* 0.06 |
|  | Interactions with family/friends^a^ |  |  |  |  |  |  |  |  |
|  | Yearly | 1.10 (0.56, 2.14) | 13.41 | 0.11 | *z =* 1.30, *p =* 0.19 | 1.39 (0.36, 5.34) | 49.72 | 0.59 |  |
|  | Monthly | 0.87 (0.74, 1.03) | 0.00 | 0.00 | *z =* 0.73, *p =* 0.46 | 0.92 (0.74, 1.15) | 14.95 | 0.01 | *z =* 1.53, *p =* 0.13 |
|  | Weekly | 0.86 (0.71, 1.04) | 5.11 | 0.01 | *z =* 1.41, *p =* 0.16 | 0.87 (0.70, 1.10) | 14.05 | 0.01 | *z =* 1.49, *p =* 0.13 |
|  | **High degree of Social Support** | **0.83 (0.71, 0.97)** | 0.00 | 0.00 | *z =* -0.06, *p =* 0.95 | 0.88 (0.75, 1.04) | 0.00 | 0.00 |  |
|  | Having a confidante | 0.91 (0.72, 1.15) | 72.04 | 0.04 | *z =* -1.67, *p =* 0.10 | 0.91 (0.73, 1.13) | 38.51 | 0.01 | *z =* -1.71, *p =* 0.09 |
|  | High relationship Satisfaction | 0.95 (0.62, 1.46) | 0.00 | 0.00 | *z =* 0.01, *p =* 1.00 | 0.99 (0.63, 1.55) | 0.00 | 0.00 | *z =* 0.41, *p =* 0.69 |
|  | **Never feeling lonely** | **0.62 (0.47, 0.82)** | 0.01 | 0.00 | *z =* 0.48, *p =* 0.63 | 0.74 (0.54, 1.02) | 0.00 | 0.00 | *z =* 0.22, *p =* 0.83 |
| Dementia | **Married/in a relationship** | **0.69 (0.49, 0.98)** | 26.01 | 0.06 | *z =* 1.39, *p =* 0.16 | **0.67 (0.47, 0.95)** | 26.80 | 0.04 | *z =* -0.20, *p =* 0.85 |
|  | Living with others | 1.01 (0.86, 1.19) | 0.00 | 0.00 | *z =* -0.09, *p =* 0.93 | 1.01 (0.85, 1.21) | 0.00 | 0.00 | *z =* -1.35, *p =* 0.18 |
|  | Community Group Engagement^a^ |  |  |  |  |  |  |  |  |
|  | Yearly | 0.98 (0.69, 1.40) | 0.00 | 0.00 | *z =* -0.34, *p =* 0.73 | 0.97 (0.67, 1.40) | 0.00 | 0.00 | *z =* -0.15, *p =* 0.88 |
|  | Monthly | 0.81 (0.57, 1.14) | 0.00 | 0.00 | *z =* -0.40, *p =* 0.69 | 0.83 (0.58, 1.20) | 0.00 | 0.00 | *z =* -0.08, *p =* 0.93 |
|  | Weekly | 0.76 (0.54, 1.07) | 0.00 | 0.00 | *z =* -0.02, *p =* 0.99 | 0.75 (0.52, 1.08) | 0.00 | 0.00 | *z =* -0.56, *p =* 0.58 |
|  | Interactions with family/friends^a^ |  |  |  |  |  |  |  |  |
|  | Yearly | 0.88 (0.53, 1.45) | 0.00 | 0.00 | *z =* 0.89, *p =* 0.37 | 0.81 (0.43, 1.52) | 0.00 | 0.00 | *z =* 0.66, *p =* 0.51 |
|  | **Monthly** | **0.48 (0.35, 0.65)** | 0.00 | 0.00 | *z =* -0.64, *p =* 0.52 | **0.48 (0.33, 0.68)** | 0.00 | 0.00 | *z =* -1.31, *p =* 0.19 |
|  | **Weekly** | **0.53 (0.41, 0.67)** | 0.00 | 0.00 | *z =* -0.78, *p =* 0.43 | **0.54 (0.39, 0.76)** | 10.78 | 0.02 | *z =* -1.25, *p =* 0.21 |
|  | High degree of Social Support | 0.83 (0.47, 1.46) | 54.29 | 0.13 | *z =* -0.68, *p =* 0.50 | 1.01 (0.57, 1.78) | 62.21 | 0.11 |  |
|  | **Having a confidante** | 0.72 (0.46, 1.14) | 69.07 | 0.13 | *z =* 0.47, *p =* 0.64 | **0.70 (0.54, 0.92)** | 4.58 | 0.00 | *z =* -0.64, *p =* 0.52 |
|  | High relationship Satisfaction | 0.68 (0.27, 1.70) | 0.00 | 0.00 | *z =* 1.19, *p =* 0.23 | 0.77 (0.27, 2.19) | 0.00 | 0.00 | *z =* 0.06, *p =* 0.95 |
|  | Never feeling lonely | 0.65 (0.36, 1.17) | 40.28 | 0.12 | *z =* -1.83, *p =* 0.07 | 0.82 (0.56, 1.19) | 0.00 | 0.00 | *z =* -1.19, *p =* 0.23 |

**Note:** csHR = cause specific Hazard Ratio

# Table S23. Subgroup analyses comparing partially adjusted models for Asian versus Western cohorts

|  | **MCI** | | | | **Dementia** | | | | **Mortality** | | | |
| --- | --- | --- | --- | --- | --- | --- | --- | --- | --- | --- | --- | --- |
| **Social Connection Marker** | **Western Cohorts HR (95%CI)** | **Asian Cohorts HR (95%CI)** | **Q** | **p-value** | **Western Cohorts HR (95%CI)** | **Asian Cohorts HR (95%CI)** | **Q** | **p-value** | **Western Cohorts HR (95%CI)** | **Asian Cohorts HR (95%CI)** | **Q** | **p-value** |
| Married/in a relationship | 0.89 (0.75, 1.06) | 1.05 (0.64, 1.73) | 0.37 | 0.54 | 0.92 (0.66, 1.29) | **0.31 (0.10, 0.98)** | 3.21 | 0.07 | 0.86 (0.72, 1.02) | 0.61 (0.05, 6.82) | 0.08 | 0.78 |
| Living with others | 0.92 (0.84, 1.02) | 1.00 (0.82, 1.22) | 0.43 | 0.51 | 1.24 (0.93, 1.66) | 0.97 (0.73, 1.28) | 1.47 | 0.22 | 0.97 (0.83, 1.12) | 0.88 (0.73, 1.06) | 0.62 | 0.43 |
| Community Group Engagement |  |  |  |  |  |  |  |  |  |  |  |  |
| Yearly | - | - | - | - | - | - | - | - | - | - | - | - |
| Monthly | 1.18 (0.91, 1.53) | 0.70 (0.35, 1.41) | 1.85 | 0.17 | 0.74 (0.47, 1.16) | 0.63 (0.18, 2.25) | 0.05 | 0.81 | **0.64 (0.50, 0.82)** | 0.53 (0.16, 1.79) | 0.09 | 0.77 |
| Weekly | 0.94 (0.64, 1.36) | 0.52 (0.21, 1.26) | 1.44 | 0.23 | 0.68 (0.43, 1.10) | 1.31 (0.39, 4.37) | 0.95 | 0.33 | **0.54 (0.41, 0.70)** | 0.80 (0.20, 3.31) | 0.30 | 0.58 |
| Interactions with family/friends |  |  |  |  |  |  |  |  |  |  |  |  |
| Yearly | 0.83 (0.56, 1.22) | 1.56 (0.10, 25.01) | 0.20 | 0.66 | 0.92 (0.51, 1.67) | 2.46 (0.14, 44.04) | 0.43 | 0.51 | - | - | - | - |
| Monthly | 0.80 (0.48, 1.34) | **0.82 (0.68, 1.00)** | 0.01 | 0.93 | **0.37 (0.22, 0.64)** | **0.55 (0.36, 0.83)** | 1.23 | 0.27 | 0.90 (0.56, 1.45) | **0.76 (0.59, 0.98)** | 0.38 | 0.54 |
| Weekly | 0.86 (0.62, 1.20) | **0.80 (0.68, 0.94)** | 0.19 | 0.67 | **0.43 (0.19, 0.99)** | **0.54 (0.40, 0.73)** | 0.27 | 0.60 | 0.88 (0.60, 1.28) | **0.62 (0.51, 0.76)** | 2.52 | 0.11 |
| High degree of Social Support | 0.86 (0.67, 1.11) | **0.81 (0.66, 0.99)** | 0.16 | 0.69 | 1.33 (0.73, 2.42) | **0.67 (0.48, 0.94)** | 3.78 | 0.05 | **0.74 (0.59, 0.93)** | 0.84 (0.66, 1.06) | 0.58 | 0.45 |
| Having a confidante | 0.86 (0.63, 1.17) | 0.92 (0.77, 1.09) | 0.14 | 0.71 | 0.70 (0.38, 1.27) | **0.64 (0.48, 0.85)** | 0.07 | 0.80 | 0.92 (0.74, 1.14) | **0.78 (0.64, 0.94)** | 1.28 | 0.26 |
| High relationship Satisfaction | - | - | - | - | - | - | - | - | - | - | - | - |
| Never feeling lonely | **0.59 (0.47, 0.74)** | 0.96 (0.46, 1.99) | 1.53 | 0.22 | 0.72 (0.49, 1.06) | **0.23 (0.06, 0.98)** | 2.24 | 0.13 | 1.00 (0.80, 1.25) | 0.63 (0.26, 1.53) | 0.96 | 0.33 |

**Note.** Estimates for Asian (CLAS, KLOSCAD, LRGSTUA, SLAS) and Western (The H70 Study, HELIAD, LEILA, MYHAT, PREHCO, ELSA, MAS) cohorts. The Q and p-values indicate whether the estimates were significantly different in Asian cohorts, compared to Western cohorts. There was insufficient data to run the analyses for yearly community group engagement and each outcome, high relationship satisfaction and each outcome, and yearly interactions with family/friends and mortality risk.

# Figure S1. Funnel plot for Mild Cognitive Impairment – Fully-adjusted models

**
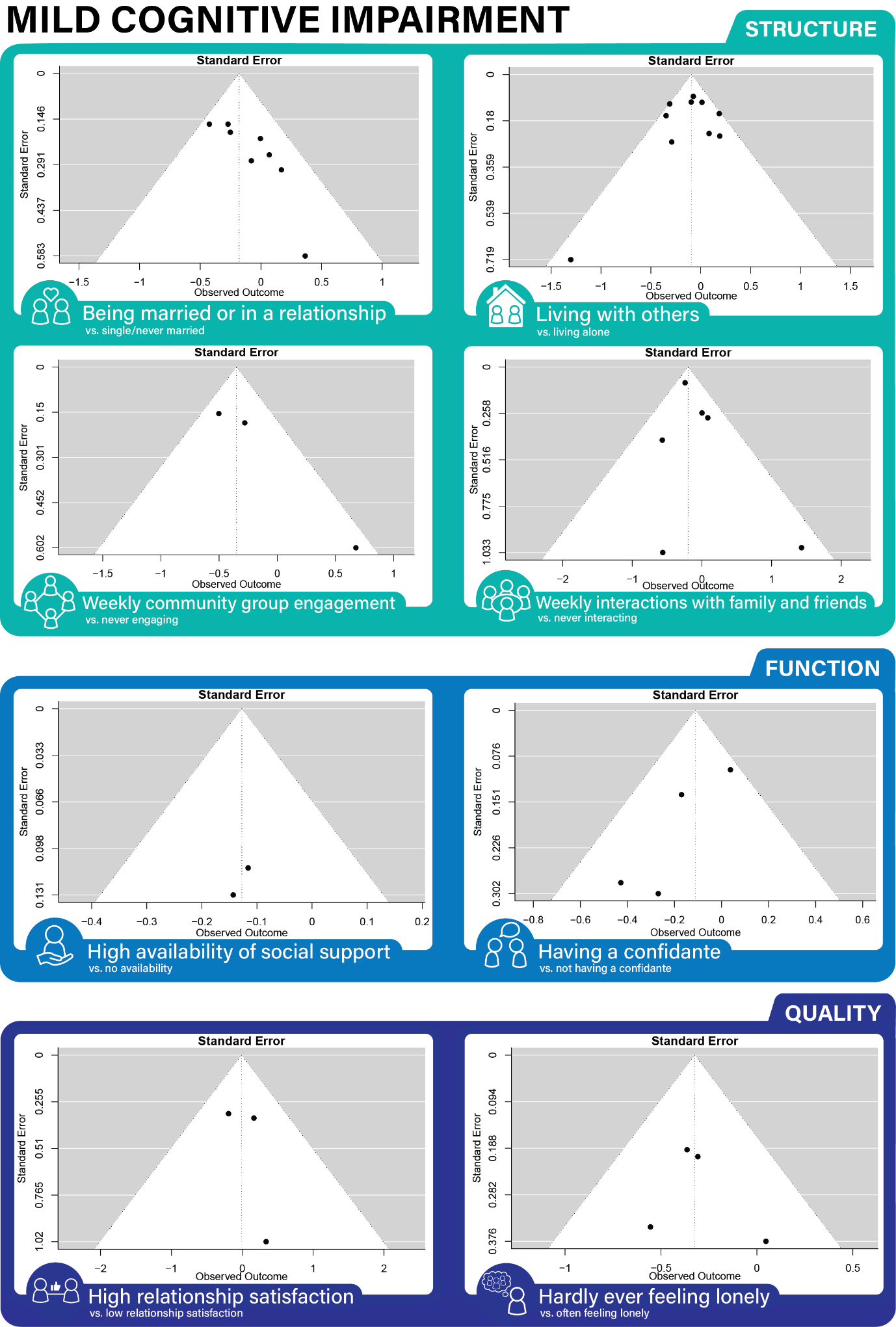
**

# Figure S2. Funnel plot for Dementia – Fully-adjusted models

**
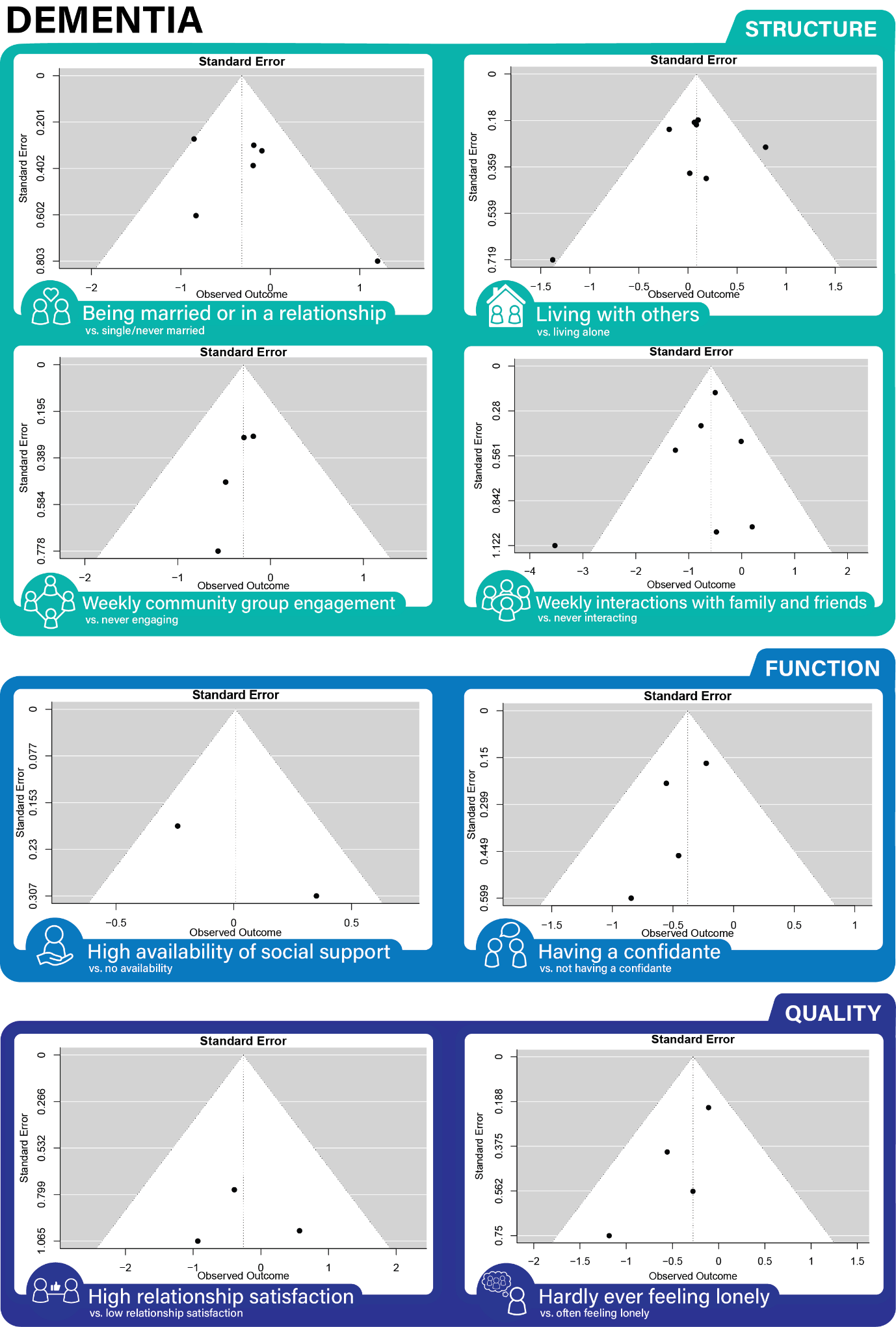
**

# Figure S3. Funnel plot for Mortality– Fully-adjusted models

**
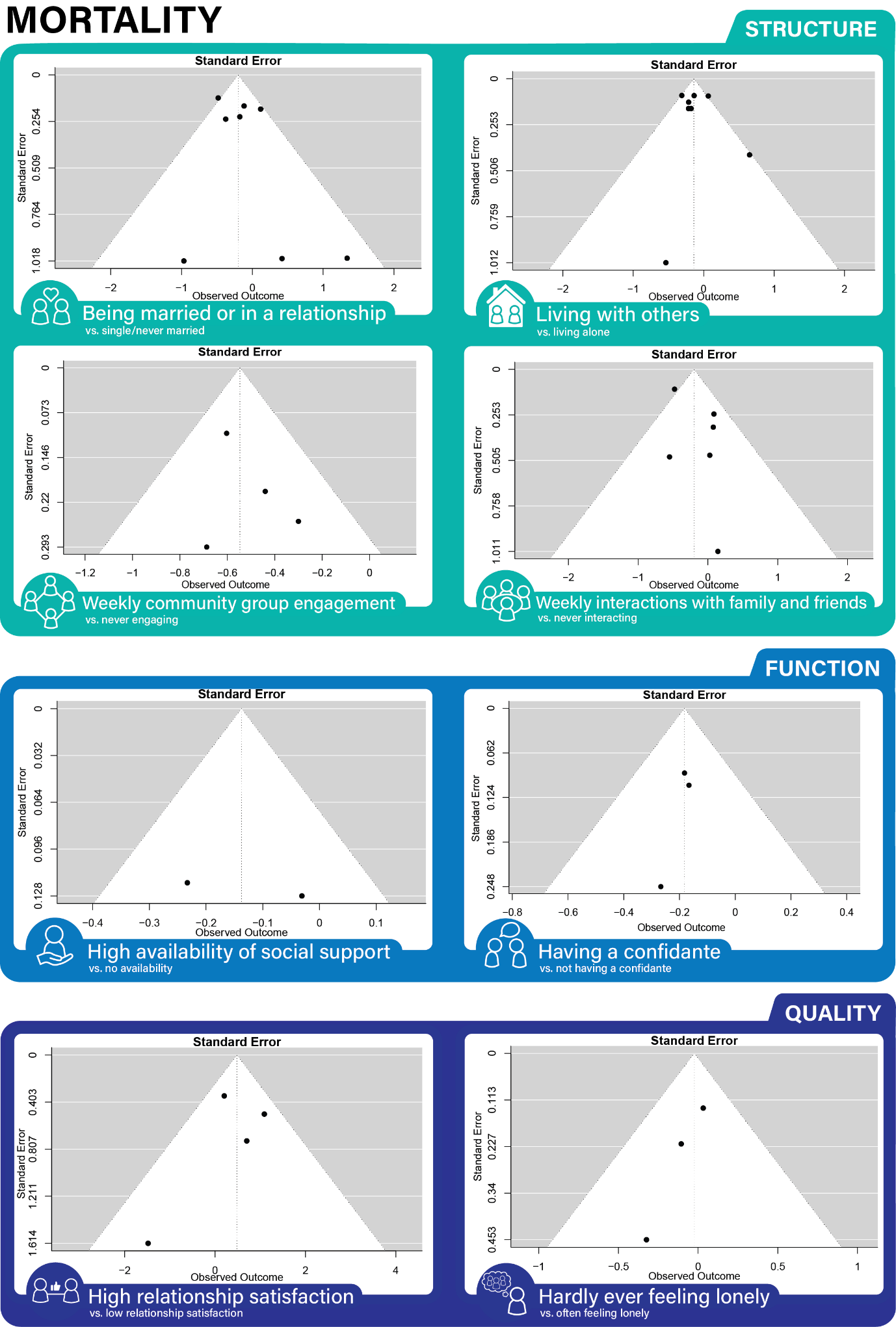
**
